# DNA–Peptide Crosslinks Placed at Codon 249 of the *p53* Tumor Suppressor Gene Induce G to A Transitions in Human Cells

**DOI:** 10.1002/cbic.70507

**Published:** 2026-08-21

**Authors:** Priscilla Yawson, Jan Henric T. Bacurio, Honnaiah Vijay Kumar, Natalia Y. Tretyakova, Ashis K. Basu

**Affiliations:** ^1^ Department of Chemistry University of Connecticut Storrs Connecticut USA; ^2^ Department of Medicinal Chemistry and the Masonic Cancer Center University of Minnesota Minneapolis Minnesota USA

**Keywords:** DNA damage, DNA–protein crosslinks, mutations, polymerase bypass, tumor suppressor gene

## Abstract

Anticancer nitrogen mustard drugs including cyclophosphamide, mechlorethamine and chlorambucil induce covalent DNA–protein cross‐links (DPCs) by sequentially alkylating the N7 position of guanine in DNA and amino acid residues within proteins. In cells, DPCs are proteolytically processed to yield corresponding peptide‐DNA cross‐links (DpCs), allowing polymerase bypass. In this study, site‐specific, hydrolytically stable DNA lesions resembling nitrogen mustard‐induced DpCs were generated via oxime ligation of an 11‐mer peptide derived from histone H4 (NH_2_–GGGK*GLGKGGA, K*=oxy‐lysine) to 7‐deaza‐7‐(2,3‐dihydroprop‐1‐yl)‐dG (DHP) in DNA. DNA sequence was derived from codon 249 of the *p53* tumor suppressor gene, a common mutational hotspot in tumors. Translesion synthesis (TLS) experiments of DpC containing plasmids in human HEK 293T cells revealed that 7‐deaza‐DpC lesion was bypassed most efficiently in wild‐type cells (83%) with lower efficiency in Pol ι KO (53%), Pol η KO (59%), and Pol κ KO (63%) cells, with Pol ζ KO unaffected (76%). Mutation frequency for polymerase bypass of 7‐deaza‐DpC (~9%) was significantly higher than for 7‐deaza‐DHP‐dG control (~5%). Pol η and Pol ι knockouts led to increased error rates, suggesting they play a role in error‐free bypass of DpCs. In contrast Pol ζ deficiency reduced mutation rates, implying that it plays a role in error‐prone bypass.

## Graphical abstract

DNA‐peptide cross‐links (DpCs) are ubiquitous DNA lesions that form when cellular proteins become covalently trapped on DNA strands and are processed by proteases. DpCs are hypothesized to play a role in antitumor activity of chemotherapeutic drugs and to contribute to aging. In this work, model DpC lesions structurally analogous to N7‐guanine DpCs were synthesized via oxime ligation and introduced into human cells to evaluate their effects on DNA replication and to identify DNA polymerases that enable their bypass.

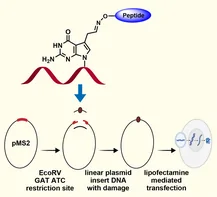

## 1 Introduction

DNA–protein crosslinks (DPCs) covalently trap proteins on DNA strands, severely obstructing DNA replication and transcription [1]. DPCs play an important role in biological activity of chemotherapeutic agents such as antitumor nitrogen mustards [2]. These lesions, if left unrepaired, trigger replication fork stalling, mutagenesis, senescence, and cell death [3]. Novel nitrogen mustard analogs that specifically induce DPCs in the absence of other types DNA damage induce toxicity in tumor cell lines [4]. Cellular repair of DPCs is initiated by proteolysis of the crosslinked proteins by Spartan metalloprotease and/or the proteasome to generate the corresponding DNA‐peptide lesions (DpCs) [5, 6]. DpCs can be bypassed by DNA polymerases in error‐free or error‐prone manner. Mutagenic bypass of DpCs can contribute to mutations and tumor resistance by inducing polymerase errors [7, 8, 9, 10].

Replication of damaged DNA in mammalian cells is mediated by a network of specialized translesion synthesis (TLS) polymerases that differ in substrate specificity, catalytic fidelity, and biological functions. Rather than acting interchangeably, individual TLS polymerases are recruited in a lesion‐dependent manner, with polymerase η (Pol η), polymerase ι (Pol ι), and polymerase κ (Pol κ) frequently functioning as nucleotide inserter polymerases, whereas polymerase ζ (Pol ζ) primarily extends from nucleotides incorporated opposite damaged bases [11, 12, 13]. Because the identity of the polymerase recruited during lesion bypass can profoundly influence both TLS efficiency and mutational outcome, these enzymes are key determinants of lesion‐specific mutagenesis [12, 14]. However, the polymerase requirements and mutagenic consequences of bypassing N7‐linked guanine‐derived DNA‐peptide lesions in mammalian cells remain poorly understood.

The *p53* tumor suppressor gene is referred to as the “guardian of the genome,” due to its crucial role in regulating cell cycle progression, apoptosis, and DNA repair in response to genotoxic stress [15]. *P53* mutations are among the most common genetic alterations in human cancers, with over 50% of tumors harboring at least one defective allele [16]. Many of these mutations occur within the DNA‐binding domain, impairing the ability of the TP53 protein to activate transcription of its downstream targets and compromising its tumor suppressive functions [17]. The *p53* codon 249 is a well‐documented mutational hotspot. Codon 249 mutations are induced by many environmental carcinogens such as aflatoxin B1 (AFB1) and by endogenous DNA damage [18, 19]. The characteristic G→T transversion at this site is a signature mutation commonly observed in hepatocellular carcinoma (HCC) [19]. Additionally, *p53* codon 249 mutations frequently lead to a substitution of arginine with serine (R249S) within the loop–sheet–helix motif of the P53 protein critical for DNA binding, producing a protein with impaired tumor suppressor function and potential gain‐of‐function oncogenic properties [20].

Given the functional importance of the *p53* gene and the adverse outcomes of codon 249 mutations, genetic integrity of this locus is critical for preventing malignant transformation. Lesions that form at or near *p53* codon 249—including bulky adducts, oxidative modifications, and DNA–protein crosslinks—pose a significant risk of introducing mutations that compromise p53 protein function. In this context, DNA–protein and DNA–peptide crosslinks are of particular interest because they strongly impede DNA replication and repair, often leading to mutagenic outcomes [1, 21]. Understanding how cells tolerate or repair these lesions and how they influence mutation spectra at critical sites such as *p53* codon 249 is essential for elucidating the molecular events driving tumorigenesis.

The most common site on DNA that participates in DNA‐protein cross‐linking mediated by nitrogen mustards and other simple *bis*‐electrophiles is the N7 position of guanine; however, such adducts are difficult to study because of their labile nature. Alkylation at the N7 position of 2′‐deoxyguanosine creates a positive charge at the affected nucleobase, destabilizing the N–glycosidic bond and leading to spontaneous depurination and abasic site formation. Scharer et al. have developed a synthetic methodology to replace the N‐7 atom of guanine with a carbon, resulting in hydrolytically stable model N7‐alkylguanine lesions. We have adopted this strategy, along with the use of unnatural amino acids (oxo‐Lys), to develop a method for synthesis of site specific, hydrolytically stable model DpC and DPC lesions [22, 23].

Polymerase bypass studies of model DNA‐protein (DPC) and DNA–peptide crosslinks (DpCs) have provided valuable insight into how such lesions interact with the cellular replication machinery [24, 25]. These studies have revealed that while all DPC adducts are strongly blocking [7], DpCs can be bypassed via translesion synthesis (TLS) in error free or error prone manner depending on their chemical structure, polymerase identity, and local sequence environment [7, 26, 27]. By probing the mutagenic properties of structurally defined DpC lesions at *p53* codon 249, such research deepens our understanding of lesion‐specific mechanisms of mutagenesis and highlights potential vulnerabilities in genome maintenance pathways.

## 2 Results and Discussion

The main goal of this study was to investigate the effects of DNA–peptide crosslinks on DNA replication in living cells using lesion‐bearing plasmids. By placing the 7‐deaza–DNA–peptide crosslinks (7‐deaza‐DpC) at codon 249 of the *p53* tumor suppressor gene, which is frequently mutated in human cancer [28, 29, 30], we aimed to determine how these types of lesions influence DNA replication efficiency and accuracy in human cells, along with characterizing their mutational outcomes and identifying TLS polymerases involved in their bypass.

### 2.1 Synthesis of Model DpC

**SCHEME 1:**
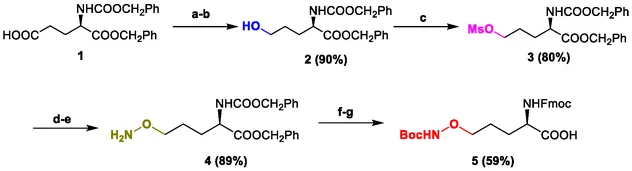
Synthesis of protected oxy‐lysine. (a) Ethylchloroformate, N‐methylmorpholine, THF, −10 °C; (b) NaBH_4_, MeOH, 0 °C; (c) methanesulfonylchloride, Et_3_N, DCM, °C; (d) N‐hydroxypthalimide, DBU, DMF, 0 °C‐RT; and (e) methylhydrazine, DCM, 0 °C; (f) Boc_2_O, Et_3_N, THF, rt; (g) H_2_/Pd/C, rt, FmocOsu, NaHCO_3_, dioxane, H_2_O, rt.

To access the oxy‐Lys building block for subsequent polypeptide assembly, glutamic acid derivative **1** (Scheme 1) was subjected to homologation via chain extension, followed by reduction with sodium borohydride (NaBH_4_) to afford the corresponding primary alcohol **2**. The resulting alcohol was then converted to mesylate **3** under standard conditions using methane sulfonyl chloride (MsCl) and triethylamine as a base. Nucleophilic substitution of **3** with N‐hydroxyphthalimide in the presence of DBU furnished the phthalimide‐protected oxyamine intermediate. Subsequent hydrazinolysis cleanly removed the phthalimide group to yield the free oxyamine **4** in an overall yield of 40% over two steps (Scheme 1) [31]. This route provides a concise and efficient entry to the oxy‐Lys motif, compatible with further coupling strategies [23]. oxy‐Lys containing peptides with 11 amino acids in length was prepared by standard solid phase peptide synthesis and structurally characterized by HPLC‐ESI‐MS [23] (Table 1). Detailed synthetic procedures and structural characterization details are given in Scheme S1, Supporting methods, and Figures S1–S6.

**TABLE 1:** Synthetic ODNs and polypeptides prepared for the conjugation studies. G* = 7‐deaza‐7‐(2‐oxoethyl)‐2′‐deoxyguanosine (dG), K* = oxy‐Lys^a^.

Compound	Sequences	*m*/*z* calc.	*m*/*z* obs.
Peptide	NH_2_‐GGG KGL GK*G GA	859.71	860.41
DNA	GGC ATG AAC CGG AG*G CCC ATC	6466.51	6465.11
DpC	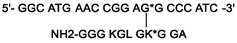	7326.22	7325.00

a Peptide was measured in +ve and DNA and DPCs are in −ve mode.

**SCHEME 2:**
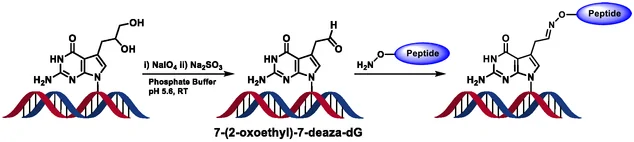
DPC generation at the C7 position of 7‐deaza‐dG via oxime ligation.

Site‐specifically modified DNA oligodeoxynucleotide (ODN) containing 7‐deaza‐7‐(2,3‐dihydroprop‐1‐yl)‐dG at *p53* codon 249 (5′‐GGC ATG AAC CGG AG*G CCC ATC‐3′, G* = 7‐deaza‐dG) were prepared by solid phase synthesis starting with the corresponding nucleoside phosphoramidites [32]. The ODN was subsequently subjected to periodate oxidation using sodium periodate (NaIO_4_) to generate 7‐deaza‐7‐(2‐oxoethyl)‐dG (Scheme 2). DNA containing site‐specific 7‐deaza‐7‐(2‐oxoethyl)‐2′‐deoxyguanosine (dG) was conjugated to oxylysine (oxy‐Lys, K*)‐containing peptide (NH_2_‐GGG KGL GK*G GA‐CO_2_H) via oxime ligation [23]. The reactions were performed in phosphate buffer (pH 5.6) at room temperature, facilitating regioselective condensation between the aldehyde moiety of 7‐deaza‐7‐(2‐oxoethyl)‐dG and the aminooxy functionality of the modified peptide. This bioorthogonal reaction yielded stable DNA–peptide cross‐link (DpC) under mild aqueous conditions (Scheme 2). The resulting DpC conjugates (7‐deaza‐DpC) were purified by gel electrophoresis and characterized by ESI MS as described previously [10].

### 2.2 Replication of DpC and 7‐Deaza DHP dG in HEK 293T Cells

**FIGURE 1:**
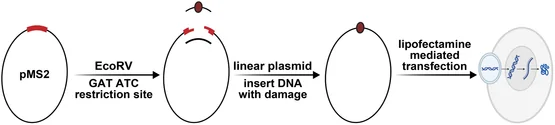
Generation and cellular replication of DpC‐containing pMS2 plasmid. A lesion‐containing oligonucleotide is ligated into EcoRV‐linearized pMS2 to generate a circular DNA construct, which is transfected into HEK 293T cells for replication. Replicated plasmids are then harvested and purified for analysis.

DpC‐containing oligonucleotide 5′‐GGC ATG AAC CGG AG*G CCC ATC‐3′ (G* = 7‐deaza‐DpC) was ligated into EcoRV‐linearized pMS2 vector to generate a circular DNA construct (Figure 1). DpC‐containing plasmids and an unmodified control plasmid containing a different sequence were co‐transfected into human embryonic kidney cells (HEK 293T cells). The plasmid lacking DpC was also co‐transfected with the unmodified control plasmid. A homologous plasmid containing a different DNA sequence served as an internal reference to assess transfection efficiency. Additionally, we have generated plasmids containing 7‐deaza‐7‐(2,3‐dihydroprop‐1‐yl)‐dG (7‐deaza‐DHP‐dG) as an additional control missing the peptide lesion but containing the 7‐deaza‐alkyl functionality. Following a 24 h incubation period, allowing for a single round of plasmid replication, progeny DNA was recovered and subsequently used to transform *E. coli* DH10B cells. The ratio of the number of colonies derived from the lesion‐bearing plasmid to those from the unmodified control plasmid was calculated to determine the translesion synthesis (TLS) bypass efficiency.

### 2.3 Distinct Contributions of Specific TLS Polymerases to Lesion Bypass Efficiency

**FIGURE 2:**
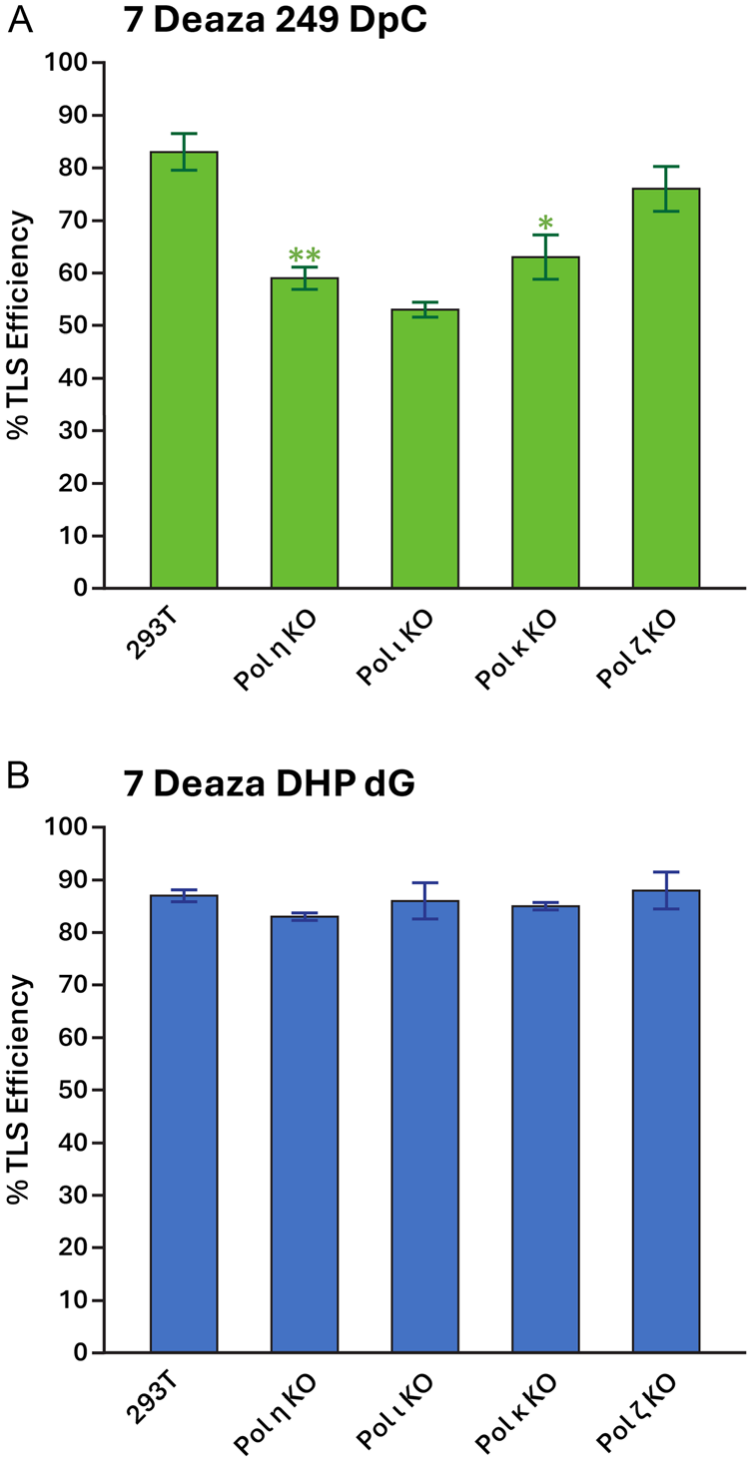
TLS efficiency of 7‐deaza‐dG crosslinked DpC (A) and 7‐deaza‐DHP‐dG control (B) in HEK 293T cells with or without TLS polymerase knockouts. Panels A (7 Deaza DpC) and B (7‐deaza‐DHP‐dG) show mean ± SD from two to three independent experiments, (*n* = 2, 3). Statistical significance was determined by two‐tailed, unpaired Student's *t*‐test (*p < 0.05, **p < 0.01).

TLS bypass efficiency of the 7‐deaza‐DpC lesions varied among different polymerase knockout (KO) cell lines compared with the wild type 293T cells. The wildtype 293T cells exhibited the highest TLS efficiency at 83%, serving as the baseline for comparison (Figure 2A). Notably, TLS efficiency was most reduced in the Pol ι KO cells (53%), suggesting a prominent role for Pol ι in DpC lesion bypass. Pol η KO and Pol κ KO cells also showed marked reductions in TLS efficiency, at 59% and 63%, respectively (Figure 2A), highlighting their contribution to bypass of DpC lesions in human cells. On the other hand, Pol ζ KO cells retained high TLS efficiency (76%) compared with other KOs, implying a potentially distinct or compensatory role for Pol ζ in this context. In contrast, TLS efficiencies for the 7‐deaza‐DHP‐dG lesion lacking the peptide crosslink were comparable across wildtype and all polymerase knockout cell lines, with values ranging narrowly between 83% and 88% (Figure 2B). Wildtype cells exhibited a TLS efficiency of 87%, establishing a high baseline for lesion bypass. The absence of individual TLS polymerases—including Pol η, Pol ι, Pol κ, and Pol ζ—did not significantly reduce TLS efficiency of 7‐deaza‐DHP, as the knockout cells maintained lesion bypass efficiencies within experimental error relative to wildtype (Figure 2B).

### 2.4 Roles of TLS Polymerase in Mutagenesis of 7‐Deaza‐DpC

**FIGURE 3:**
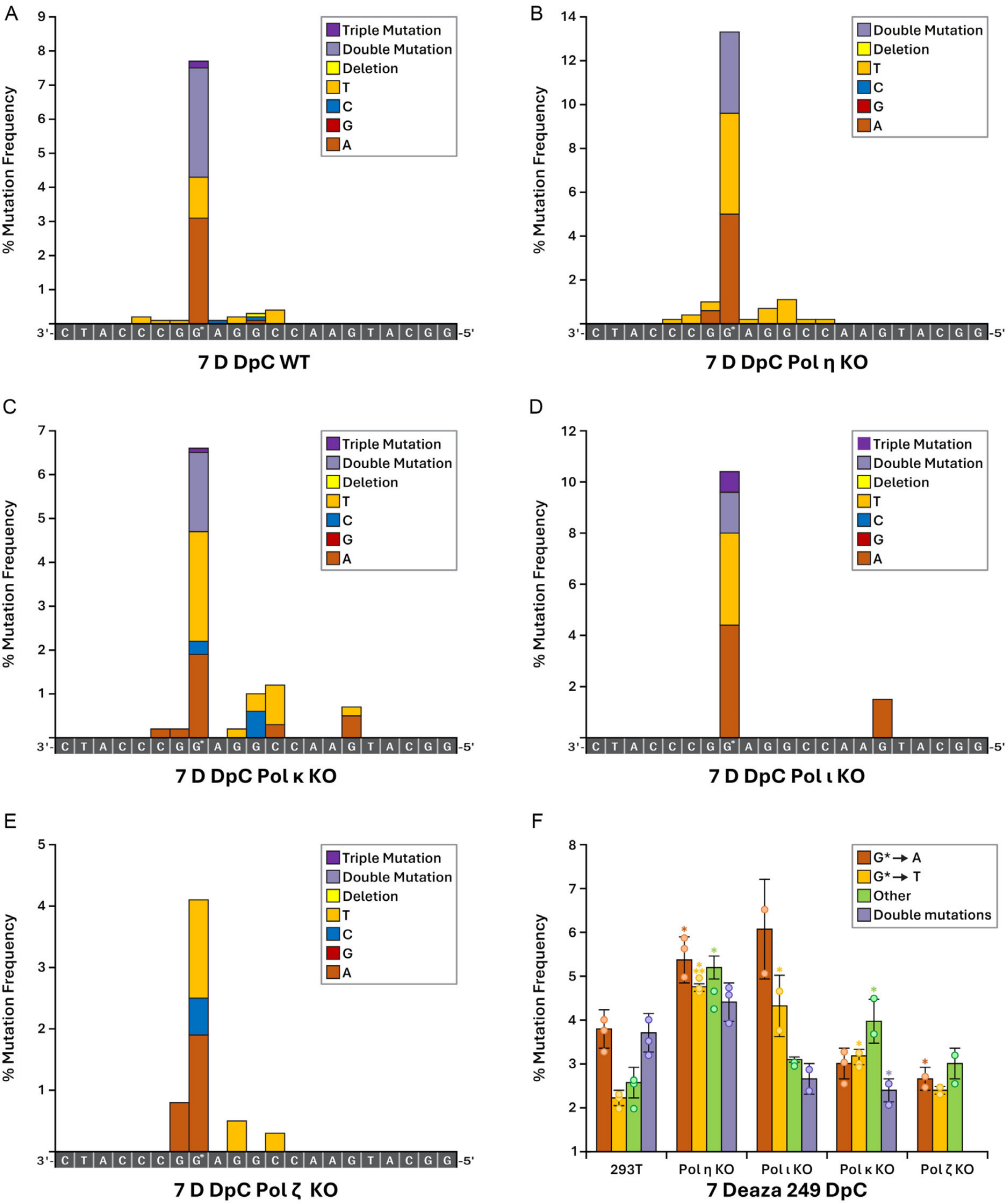
Types and frequencies of mutations induced by 7‐deaza‐DpC in the *p53* codon 249 sequence in HEK 293T cells and polymerase knockout variants. Panel A shows data from wild‐type HEK 293T cells, while Panels B–E display results from various polymerase knockout cell lines, while Panel F shows the overall summary of the results. Error bars represent mean values ± standard deviation from three to four independent experiments (*n* = 3, 4). Dots represent replicates. Statistical significance was determined by two‐tailed, unpaired Student's *t*‐test (*p < 0.05).

The mutagenic potential of 7‐deaza‐DpC was assessed in HEK 293T cells, comparing wildtype and TLS polymerase knockout cell lines. Lesion bypass in wildtype cells yielded a total mutation frequency of 9.4% ± 1.2%, with the dominant mutation being G*→A transitions (3.2%) and a comparable contribution of double mutations (3.1%). Lower levels of G*→T mutations (1.4%) and off‐target events (1.5%) were also observed (Figure 3A,F). These findings suggest that while 7‐deaza‐DpC is modestly mutagenic, and replication past the lesion in wildtype cells is accomplished via accurate or semi‐accurate TLS activity.

In contrast, Pol η knockout cells exhibited a substantial increase in total mutation frequency to 18.2% ± 1.5%, nearly double that of the wildtype. Both G*→A (5.5%) and G*→T (4.5%) mutations increased significantly, as did off‐target mutations (4.1% ± 0.3%) and double mutations (4.1% ± 0.6%) (Figure 3B,F). The low standard deviations indicate consistent results across replicates, supporting the interpretation that Pol η plays a critical role in high‐fidelity bypass of DpC lesions. Pol η loss shifts lesion bypass to more error‐prone polymerases or leads to misincorporation and multiple substitutions, indicating that Pol η is essential for suppressing both single‐nucleotide and complex mutagenesis induced by the DpC.

In the Pol κ knockout clone, the total mutation frequency (9.9% ± 0.6%) was comparable to wildtype; however, the mutation pattern shifted. G*→T transversions increased slightly to 2.5% ± 0.2%, while G*→A transitions decreased to 2.3% ± 0.4% (Figure 3C,F). A small rise in G*→C transversions (0.3% ± 0.5%) was also observed (Figure 3). Despite the minimal increase in total mutation frequency, the distribution of mutation types suggests that Pol κ influences the selection of bypass pathways, possibly favoring more accurate lesion resolution.

Pol ι‐deficient cells demonstrated a modestly elevated mutation frequency (11.6% ± 4.3%), as a result of G*→A transitions (4.7% ± 2.1%) and off‐target mutations (1.2% ± 0.9%) (Figure 3D,F). Although the average number of mutant clones was higher than for wildtype cells, large variance observed across replicates suggests heterogeneous processing of the lesion in the absence of Pol ι. The appearance of triple mutations (0.7% ± 0.8%) (Figure 3D) also indicates that Pol ι may help suppress some of the more complex replication errors. Together, these findings suggest Pol ι plays a smaller/supportive role in limiting error‐prone bypass of DpC lesions.

Pol ζ knockout cells had the lowest overall mutation frequency at 6.0% ± 0.7%, which is significantly reduced compared to wildtype. This reduction was consistent across mutation types including G*→A (2.1% ± 0.3%), G*→T (1.7% ± 0.1%), and off‐target mutations (1.8% ± 1.1%), with no double or triple mutations detected (Figure 3E,F). This suggests that in the absence of Pol ζ, the cell's ability to extend mismatched termini is impaired, reducing mutation fixation. This aligns with Pol ζ's known function as an extender polymerase during TLS and highlights its role in facilitating mutagenic bypass downstream of insertion events [33].

**FIGURE 4:**
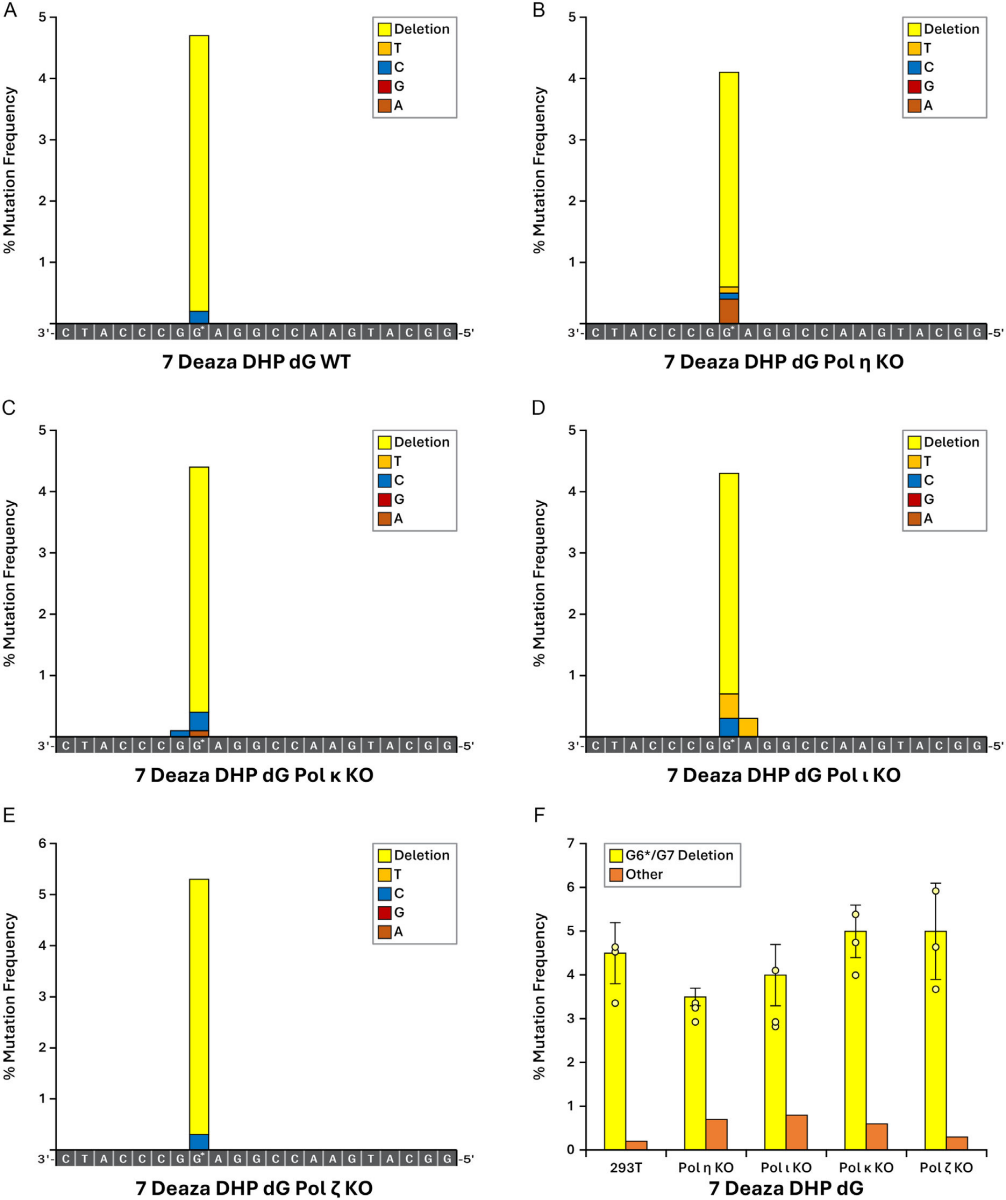
Types and frequencies of mutations induced by 7‐deaza‐DHP‐dG placed within the *p53* codon 249 in HEK 293T cells and the corresponding TLS polymerase knockout variants. Panel (A) shows data from wild‐type HEK 293T cells, while Panels (B–E) display results from various polymerase knockout cell lines, while Panel (F) shows the overall summary of the results. Error bars represent mean values ± standard deviation from three to four independent experiments (*n* = 3, 4). Dots represent replicates. Statistical significance was determined by two‐tailed, unpaired Student's *t*‐test.

On the other hand, mutation profile of 7‐deaza‐DHP‐dG monoadduct in all cell clones revealed a low overall mutational burden, with total mutation frequencies ranging from 4.2% ± 0.6% (Pol η knockout) to 5.5% ± 0.1% (Pol κ knockout) (Figure 4). Across all polymerase‐deficient and wildtype backgrounds, the predominant lesion‐associated event was G* deletion, comprising most of the observed mutations (Figure 4A–F). This suggests that structural instability at or near the DpC lesion site, leading to deletions, rather than base substitution, as the major outcome of 7‐deaza DHP dG incorporation into plasmid DNA. In contrast, base substitutions (G*→A/T/C) were rare (typically ≤0.4%) and exhibited minimal variability (Figure 4).

## 3 Conclusions

Collectively, our data demonstrate that plasmids containing 7‐deaza‐DpC lesion placed within codon 249 of the *p53* gene and conjugated to histone H4 peptide NH_2_‐GGG KGL GK*G GA‐CO_2_H via oxime ligation are replicated with high efficiency in human HEK 293T cells. TLS bypass of these DpC lesions was only modestly reduced by deficiency in TLS polymerases. This is in contrast with previous report of inefficient bypass of a structurally analogous DpC adduct placed within the AG*T sequence context and conjugated to a 10‐mer peptide derived from c‐Myc protein (Ac–Glu–Gln–Lys–Leu–Ile–Ser–Glu–Glu–Asp–Leu–CONH_2_) [27], suggesting that DNA sequence context and or peptide sequence have a profound effect on translesion bypass of DpC. Our previous studies revealed that neighboring bases [34] and the nature of the peptide [9] can affect replication past DpC lesions. Additionally, the structure of the cross‐link (e.g., imine vs. oxime linkage) can alter how the replication machinery interacts with the lesion [9]. Importantly, this difference has direct relevance to *p53* mutagenesis: Efficient yet error‐prone bypass of DpC lesions formed at codon 249 could promote mutagenesis and interfere with tumor‐suppressive function of the *p53* gene.

Although polymerase bypass of 7‐deaza‐DpC lesions placed at *p53* codon 249 was efficient, it was not error‐free. Replication across this lesion in wild‐type HEK 293T cells resulted in mutagenesis (~9%), dominated by targeted G→A transitions and G→T transversions and accompanied by double mutations. Our observation of G→T transversions at the third position of *p53* codon 249 is particularly noteworthy given the predominance of such base changes at p53 codon 249 mutational hotspot (AGG→AGT, resulting in an Arg→Ser substitution). Thus, even though the DpC lesions are efficiently bypassed, replication fidelity is compromised, giving rise to biologically significant mutations that could phenocopy oncogenic *p53* variants observed in human tumors.

The predominance of G→A transitions observed in this study contrasts with the well‐established G→T transversion induced by aflatoxin B1 at TP53 codon 249. This difference suggests that structurally distinct guanine adducts can generate unique mutational outcomes despite occurring at the same genomic locus. Such lesion‐specific mutation spectra highlight the importance of adduct chemistry and DNA polymerase selection in determining mutational consequences.

TLS polymerase knockout experiments reveal important trends directly relevant to *p53* mutagenesis (Figure 3). The loss of Pol η led to a nearly twofold increase in mutagenesis, confirming its role in error‐free bypass of 7‐deaza‐DpC. In contrast, deletion of Pol ζ markedly reduced the overall mutation frequency, consistent with its known function as a mutagenic extender polymerase. Knockouts of Pol κ and Pol ι induced only minor changes in bypass efficiency and mutation outcomes. Compared with the findings of Pande et al. [27], our results reveal a key difference: In their study, Pol κ played a more pronounced role in suppressing mutagenesis, and the absence of Pol η led to an even greater increase in mutation frequency. Together, these findings suggest that DNA sequence and chemical structure of the 7‐deaza‐DpC lesion modulate polymerase selection and fidelity during TLS, thereby leading to different mutational outcomes. The interplay between Pol η and Pol ζ may determine whether bypass proceeds through an error‐free or error‐prone pathway, ultimately influencing whether *p53* gene retains its tumor‐suppressive capacity.

In contrast to our results for DpC containing plasmids, our results also show that replication across 7‐deaza‐DHP‐dG lesions missing a cross‐link to a peptide is not dependent on any single TLS polymerase. Instead, the lesion appears to be bypassed efficiently by multiple polymerases or by the replicative machinery itself. The lack of a pronounced decrease in TLS efficiency 7‐deazaG monoadducts in any knockout background contrasts with our observations for DpC lesions described above. Thus, 7‐deaza‐DHP‐dG is likely a nonblocking lesion that is efficiently tolerated during replication, with minimal reliance on specialized TLS polymerases.

Taken together, these results indicate that the local DNA sequence and peptide identity within 7‐deaza‐G DpC modificationalters the structural properties of the lesion in a way that facilitates polymerase engagement and reduces mutagenesis. The distinct replication outcome for this lesion, compared to the imine DpC studied by Pande et al. [27] and DpC lesions attached to the C‐5 position of cytosine described by Bacurio et al. [24], underscores the influence of lesion structure on TLS outcomes. From a biological perspective, this is particularly significant in the context of *p53*, where codon 249 serves as a sentinel site for mutagenic processes linked to carcinogen exposure and replication stress. Efficient but imperfect TLS across lesions at this site could represent a double‐edged sword—maintaining replication continuity while seeding mutations that disable p53 function. These findings provide new mechanistic insights into how DpC lesion structure dictates polymerase choice and fidelity at functionally critical genomic sites, advancing our understanding of potential roles of endogenous and chemotherapy‐induced DNA–peptide crosslinks in *p53*‐driven tumorigenesis.

### 3.1 Limitations of the Study

It should be noted that the present study employed a single‐stranded plasmid‐based replication system in which the lesion is encountered in the absence of an opposing complementary strand. In double‐stranded DNA, additional factors, including lesion‐induced perturbations of duplex structure, accessibility to DNA repair factors, replication fork architecture, and the recruitment and coordination of translesion synthesis polymerases, may influence lesion processing and mutagenic outcomes [12, 13, 35]. Consequently, the bypass efficiencies and mutation spectra observed in the current system may not fully recapitulate those occurring in chromosomal double‐stranded DNA.

## 4 Experimental Section

### 4.1 Materials

Solvents and reagents used for synthesis and purification were purchased from Sigma‐Aldrich (St. Louis, MO, USA) or Thermo Fisher Scientific (Waltham, MA, USA), unless otherwise specified. Nucleoside phosphoramidites, solid supports, and all other reagents required for the solid‐phase synthesis of DNA were obtained from Glen Research (Sterling, VA, USA). Synthetic DNA oligodeoxynucleotides were synthesized by solid phase synthesis using an ABI 394 DNA synthesizer (Applied Biosystems, CA).

### 4.2 Synthesis of Protected‐Aminooxy Lysine (oxy‐Lys)

**SCHEME 3:**
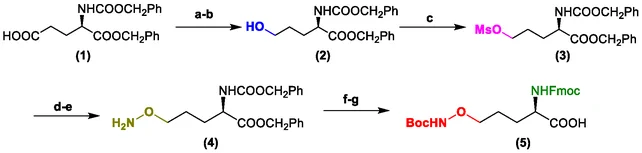
Synthesis of protected oxy‐lysine. (a) Ethylchloroformate, *N*‐methylmorpholine, THF, −10 °C; (b) NaBH_4_, MeOH, 0 °C; (c) methanesulfonylchloride, Et_3_N, DCM, °C; (d) *N*‐hydroxypthalimide, DBU, DMF, 0 °C‐RT. (e) methylhydrazine, DCM, 0 °C; (f) Boc_2_O, Et_3_N, THF, rt; and (g) H_2_/Pd/C, rt, FmocOsu, NaHCO_3_, dioxane, H_2_O, rt.

Protected‐aminooxy lysine (oxy‐Lys) was synthesized as shown in Scheme 3.

### 4.3 Synthesis of Benzyl (R)‐2‐(benzyloxy)carbonyl)amino)‐5‐Hydroxypentanoate (2)

This compound was synthesized as reported in literature [36]. The spectroscopic and analytical data were consistent with those reported in the literature. ^1^H NMR (CDCl_3_, 500 MHz): *δ* 7.26 (s, 10H), 5.40–5.41 (d, 1H, J = 6.5 Hz), 5.06–5.13 (m, 2H), 5.03–5.5.05 (m, 2H), 4.37–4.41 (m, 1H), 3.54 (t, 1H, J = 6.1 Hz), 1.84–1.1.91 (m, 1H), 1.67–1.74 (m, 1H), 1.42–1.55 (m, 1H). ^13^C NMR (CDCl_3_, 125 MHz): δ 172.1, 155.8, 140.9, 136.1135.2, 128.6, 128.5, 128.5, 128.3, 128.2, 128.1, 127.6, 126.967.4, 67.2, 67.1, 67.0, 65.3, 62.0, 60.4, 59.2, 53.6, 49.6, 29.3, 28.0, 25.5, 21.0, 20.9, 14.2.5.

### 4.4 Synthesis of Benzyl (R)‐2‐(((benzyloxy)carbonyl)amino)‐5‐((methylsulfonyl)oxy)pentanoate (3)

This compound was synthesized as reported in literature [31]. The spectroscopic and analytical data were consistent with those reported in the literature. ^1^H NMR (CDCl_3_, 500 MHz): *δ* 7.28 (s, 10H), 5.27–5.29 (d, 1H, *J* = 7.5 Hz), 5.05–5.14 (m, 2H), 5.03 (s, 2H), 4.38–4.39 (d, 1H, *J* = 5 Hz), 4.12 (s, 2H), 2.88 (s, 3H), 1.93–1.94 (d, 1H, *J* = 5.5 Hz), 1.66–1.73 (m, 1H). ^13^C NMR (CDCl_3_, 125 MHz): δ 171.7, 155.9, 136.0, 135.0, 128.7, 128.6, 128.5, 128.4, 128.2, 128.1, 68.8, 67.4, 67.1, 60.4, 53.2, 50.8, 37.3, 28.9, 25.0, 21.0, 14.1.

### 4.5 Synthesis of Benzyl (R)‐2‐(((benzyloxy)carbonyl)amino)‐5‐((1,3‐dioxoisoindolin‐2yl) oxy)pentanoate (3a)

This compound was synthesized as reported in literature [31]. The characterization data matches the reported data. ^1^H NMR (CDCl_3_, 500 MHz): *δ* 7.73–7.74 (m, 2H), 7.63–7.67 (m, 2H), 7.24–7.28 (m, 10H), 5.42–5.43 (d,*J* = 8 Hz, 1H), 5.12 (s, 2H), 5.02 (s, 2H), 4.39–4.43 (m, 1H), 4.11 (br s, 2H), 2.07–2.09 (m, 1H), 1.89–1.97 (m, 1H), 1.71–1.75 (m, 2H). ^13^C NMR (CDCl_3_, 125 MHz): δ 172.1, 163.7, 156.1136.4, 135.4, 134.6, 129.0, 128.7, 128.6, 128.5, 128.4, 128.2, 128.1, 123.6, 67.3, 67.1, 53.8, 28.9, 24.4.

### 4.6 Synthesis of Benzyl(R)‐5‐(aminooxy)‐2‐(((benzyloxy)carbonyl)amino) pentanoate (4)

To a stirred solution of compound **3a** (2.49 g, 4.9 mmol) in dichloromethane (60 mL) at 0 °C was added methylhydrazine (7.44 mmol, 392 μL). The reaction mixture was stirred at 0 °C for 1 h. Upon completion of the reaction, as monitored by TLC, the mixture was passed through a pad of Celite, and the filtrate was concentrated under reduced pressure. The resulting crude residue was purified by flash column chromatography to afford compound **3** (1.65 g, 89%) as a off‐white solid. TLC (silica gel, CH_2_Cl_2_/MeOH, 90:10) *R*f 0.3. ESI+ ‐MS: Exact mass calc. for [C_20_H_23_N_2_O_4_]^+^ 355.1652 [M + H]^+^, Exact mass obs: 355.1656 [M + H]^+^. ^1^H NMR (CDCl_3_, 500 MHz): *δ* 7.35 (s, 11H), 5.44–5.46 (d, *J* = 8 Hz, 1H), 5.17–5.20 (m, 2H), 5.13 (s, 2H), 4.45–4.49 (m, 1H), 3.66–3.69 (m, 2H), 3.51 (s, 1H), 1.92–1.97 (m, 1H), 1.74–1.86 (m, 1H), 1.60–1.69 (m, 1H). ^13^C NMR (CDCl_3_, 125 MHz): δ 172.2, 155.9, 136.2, 135.2, 128.6, 128.5, 128.5, 128.3, 128.2, 128.1, 74.9, 67.2, 67.0, 53.7, 50.8, 49.6, 24.0.

### 4.7 Synthesis of Benzyl (R)‐2‐(benzyloxy)carbonyl)amino)‐5(ter butoxycarbonyl) (amino)oxy) pentanoate (4a)

To a solution of compound **4** (100 mg, 0.26 mmol) in dry THF (16 mL) was added triethylamine (54 mg, 0.52 mmol) and di‐tert‐butyl dicarbonate (116 mg, 0.52 mmol) and the reaction mixture was stirred at room temperature for 3 h. After completion of the reaction (as monitored by TLC), the mixture was evaporated to dryness under reduced pressure and subjected to flash column chromatography (FC) to give compound **4a** (90 mg, 72%) as a colorless oil. TLC (silica gel, EtOAc/Hexanes, 70:30) *R*f 0.5. ESI+‐MS: Exact mass calc. for [C_25_H_31_N_2_O_6_]^+^ 455.2177 [M + H]^+^, Exact mass obs: 455.1215 [M + H]^+^. ^1^H NMR (CDCl_3_, 500 MHz): *δ* 7.23–7.27 (m, 11H), 7.11 (s, 1H), 5.49–5.51 (d, *J* = 8 Hz, 1H), 5.06–5.12 (m, 2H), 5.02 (s, 2H), 4.32–4.37 (m, 1H), 3.73 (t, 1H), 1.87–1.91 (m, 1H), 1.75–1.79 (m, 1H), 1.55–1.59 (m, 2H). ^13^C NMR (CDCl_3_, 125 MHz): *δ* 172.2, 157.0, 156.1, 136.2, 135.3, 128.6, 128.5, 128.3, 128.1, 81.7, 75.8, 67.1, 66.9, 53.8, 29.0, 28.1, 23.9.

### 4.8 Synthesis of (R)‐2‐(9H‐fluoren‐9‐yl)methoxy)carbonyl)amino)‐5‐(tert‐butoxycarbonyl) amino)oxy) Pentanoic Acid (5)

Solution of compound **4a** (0.6 g, 1.27 mmol) in methanol (10 mL) at room temperature was stirred with 10% Pd/C (0.270 g, 2.53 mmol) under 1 atm H_2_ for 2 h. The Pd/C was removed by filteration, and filtrate was concentrated. The residue was dissolved in dioxane/water mixture (1:1, v/v, 10 mL). To this solution was added 9‐fluorenylmethylsuccinimidyl carbonate (FmocOsu) (0.51 g, 1.51 mmol) and NaHOC_3_ (0.23 g, 2.75 mmol) and stirred for overnight at room temperature. The reaction mixture was further acidified to pH 3–4 by addition of 1 N HCl. After neutralization, the reaction solution was extracted with ethyl acetate (3× 30 mL) and the combined organic extracts were washed with water (30 mL) and brine (30 mL) and dried over anhydrous Na_2_SO_4_ and evaporated to dryness under reduced pressure. The residue was further purified by FC to obtain as viscous oil. Lyophilization from acetonitrile‐water provided off‐white solid (0.35 g, 59%). TLC (silica gel, DCM/MeOH, 90:10) *R*f 0.3. ESI+−MS: Exact mass calc. for [C_25_H_30_N_2_NaO_7_] + 493.1945 [M + Na]+, Exact mass obs: 493.1946 [M + Na]+. ^1^H NMR (CDCl_3_, 500 MHz): *δ* 7.65 (bs, 2H), 7.50 (bs, 3H), 7.28 (bs, 2H), 7.12 (bs, 2H), 4.28–4.35 ( m, 3H), 4.10 (bs, 1H), 3.76–3.78 (m, 2H), 1.94 (bs, 1H), 1.78 (bs, 1H), 1.62 (bs, 2H), 1.37 (s, 9H). ^13^C NMR (CDCl_3_, 125 MHz): *δ* 143.73, 141.25, 127.67, 127.09, 125.20, 119.92, 53.43, 47.10, 29.71, 28.22.

### 4.9 Synthesis of 7‐Deaza‐dG‐Containing DNA–Peptide Conjugates

**SCHEME 4:**
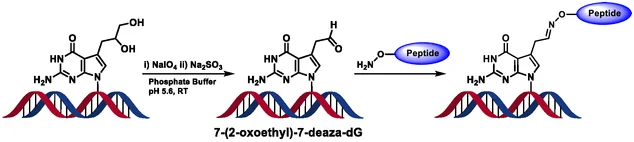
Synthesis of site‐specifically modified DNA oligodeoxynucleotide containing 7‐deaza‐7‐(2,3‐dihydroprop‐1‐yl)‐dG.

7‐Deaza‐dG‐containing DNA–peptide conjugates were prepared via oxime ligation as shown in Scheme 4:

#### 4.9.1 Step 1: Oxidation of the DNA Aldehyde Precursor

To a solution of site‐specifically modified DNA oligodeoxynucleotide (ODN) containing 7‐deaza‐7‐(2,3‐dihydroprop‐1‐yl)‐dG (200 pmol) in a microcentrifuge tube added sodium phosphate buffer (15 mM, pH 5.6; 10 μL of a 30 mM stock solution) and nuclease‐free water to a final volume of 20 μL. Sodium periodate (NaIO_4_, 10 mM final concentration; 10 μL of a 200 mM stock solution) was then added, and the reaction mixture was incubated for 6 h at 4 °C in the dark. Upon completion, the reaction was quenched by the addition of sodium sulfite (Na_2_SO_3_, 100 mM final concentration; 5 μL of a 500 mM stock solution). The reaction mixture was desalted using a NAP‐5 column, and the oxidized DNA was concentrated under reduced pressure using a Speed‐Vac to get approximately 20–25 pmol of DNA Aldehyde Precursor with 83% yield.

#### 4.9.2 Step 2: Oxime Ligation With Peptide

The obtained DNA aldehyde precursor was treated with sodium phosphate buffer (15 mM final concentration, pH 5.6; 10 μL of a 30 mM stock solution) was added to the residue, followed by the peptide (20 nmol, 100 equiv relative to DNA). Nuclease‐free water was then added to adjust the total reaction volume to 20 μL. The reaction mixture was thoroughly mixed and incubated overnight at 37 °C to allow oxime bond formation between the aldehyde‐functionalized DNA and the aminooxy‐containing peptide [23]. After completion, the reaction mixture was desalted on a NAP‐5 column, concentrated using a Speed‐Vac, and the resulting DNA–peptide conjugate (DpC, 52% yield) was characterized by LC–MS.

### 4.10 Purification

DNA samples were mixed with SDS loading dye and subjected to electrophoresis on a 10% SDS‐polyacrylamide gel to separate DpC from starting DNA and reaction byproducts. The DpC band was excised, recovered from the gel, and purified. An aliquot of the purified DpC was then end‐labeled with [γ‐^32^P] ATP and analyzed on a second 10% SDS‐polyacrylamide gel. Following electrophoresis, the gel was dried and exposed to a phosphor screen, and radiolabeled DNA was visualized by phosphor imaging to assess sample purity.

### 4.11 Reagents

All chemicals and solvents were of analytical grade and used without further purification unless otherwise stated. Restriction endonuclease EcoRV‐HF, T4 DNA ligase, T4 polynucleotide kinase, uracil DNA glycosylase, and exonuclease III were purchased from New England Biolabs. The lesion‐containing oligodeoxynucleotide used in this study had the sequence 5′‐GGCATGAACCGGAG*GCCCATC‐3′, where G* denotes 7‐deaza‐DHP‐dG covalently linked to the lysine residue (K*) of an 11‐mer peptide (NH_2_–GGGKGLGKGGA). Control oligodeoxynucleotides contained the same DNA sequence with G replaced by unmodified 7‐deaza‐DHP‐dG. All unmodified oligonucleotides, including scaffolds and probes, were purchased from Integrated DNA Technologies, and their sequences are provided in the supplementary data. [γ‐^32^P] ATP was obtained from Revvity Inc. (formerly PerkinElmer Health Sciences). Plasmid pMS2 was kindly provided by Professor M. Moriya (Stony Brook University, The State University of New York). Human embryonic kidney (HEK 293T) cells were obtained from the American Type Culture Collection (Manassas, VA) (ATCC Cat # CRL‐3216, RRID:CVCL_0063). TLS polymerase‐deficient HEK 293T cell lines (lacking hPol η, hPol κ, hPol ι, hPol ζ) were generously provided by Professor Yinsheng Wang (University of California, Riverside) and generated via CRISPR‐Cas9 genome editing [37, 38].

### 4.12 Construction and Characterization of pMS2 Vector Containing a Single 7‐Deaza‐DpC Lesion

The single lesion pMS2 vector, harboring ampicillin and neomycin resistance markers, was constructed using a site‐specific ligation strategy (Figure S1). Briefly, 5 μg of pMS2 DNA was digested with excess EcoRV‐HF at 37 °C for 5 h. The digested plasmid was annealed with a 56‐mer oligonucleotide scaffold containing four uracil bases by heating to 75 °C followed by slow cooling to room temperature and overnight incubation at 4 °C to generate a gapped circular DNA intermediate. Control and lesion‐containing oligonucleotides were phosphorylated with T4 polynucleotide kinase, annealed to the gapped vector, and ligated overnight at 16 °C. The scaffold strand was subsequently removed by digestion with uracil DNA glycosylase and exonuclease III at 16 °C for 13 h. Unligated oligonucleotides and residual salts were eliminated using Amicon Ultra‐0.5 mL centrifugal filters (100K MWCO, Millipore Sigma). Circularization efficiency was assessed by electrophoresis on a 1% agarose gel, confirming ≥40% ligation efficiency across the gap.

### 4.13 Replication in Human Embryonic Kidney (HEK 293T) and TLS Polymerase‐Deficient Cells

HEK 293T cells were cultured in Dulbecco's Modified Eagle's Medium supplemented with 4 mM L‐glutamine, 1.5 g/L sodium bicarbonate, 4.5 g/L glucose, and 10% fetal bovine serum. Cells were maintained at 37 °C in a humidified 5% CO_2_ atmosphere and used for transfection at 70%–80% confluence. For each transfection, 60 ng of lesion‐containing pMS2 and 60 ng of an internal control (IC) vector, harboring a distinct 12‐mer sequence at the ligation site, were combined with Lipofectamine (3.6% final concentration; Invitrogen) in Opti‐MEM medium (Gibco). For controls, unmodified pMS2 vectors containing 7‐deaza‐DHP‐dG at the corresponding site were co‐transfected with the IC construct at a 1:1 molar ratio. After 15 min incubation at room temperature to allow liposome complex formation, the mixtures were added to the cells. Following 20–22 h incubation at 37 °C, cells were harvested using RLT lysis buffer (Qiagen) supplemented with β‐mercaptoethanol (Sigma). The same experimental procedure was applied to TLS polymerase‐deficient cell lines.

### 4.14 Determination of Lesion Bypass Efficiencies and Mutation Analysis

Progeny plasmids were recovered using the QiaPrep Spin Miniprep Kit (QIAGEN), yielding typically 90–120 ng DNA. The recovered DNA was amplified by transformation into E. coli DH10B, and transformants were analyzed using oligonucleotide hybridization. Probes complementary to the insertion site (central probe) and to sequences flanking the ligation site (left and right probes) were used to distinguish correctly replicated sequences from mutants.

Colonies hybridizing with all three probes were scored as faithful bypass events, while those hybridizing only with the flanking probes and not with the central probe were considered putative mutants and subjected to DNA sequencing. Colonies failing to hybridize with the flanking probes were excluded from analysis. TLS efficiency was calculated as the ratio of colonies derived from lesion‐containing plasmids to those from the internal control vector.

### 4.15 Statistical Analysis

Data are presented as mean ± standard deviation (SD). The sample size (*n*) for each analysis is indicated in each figure legend. *p* values are derived from two‐tailed, unpaired Student's *t*‐test. *p* values less than 0.5 are considered statistically significant. All statistical tests were performed using GraphPad Prism software (v8).

## Funding

This study was supported by the National Institute of Environmental Health Sciences (R01‐ES023350).

## Conflicts of Interest

The authors declare no conflicts of interest.

## Supporting information

Supplementary Material

## Data Availability

The data that support the findings of this study are available from the corresponding author upon reasonable request.
