# Supplementary material for: DNA–Peptide Crosslinks Placed at Codon 249 of the p53 Tumor Suppressor Gene Induce G to A Transitions in Human Cells
Source: Chembiochem. 2026 Aug 21;27(16):e70507. doi: 10.1002/cbic.70507 (PMC13495116; doi:10.1002/cbic.70507)
Supplement: Supplementary file 1 [file CBIC-27-e70507-s001.pdf]

Supplementary Information

DNA–Peptide Crosslinks Placed at Codon 249 of the p53 Tumor Suppressor Gene Induce G to A Transitions in Human Cells

Priscilla Yawson,[a] Jan Henric T. Bacurio,[a] Honnaiah Vijay Kumar,[b,c] Natalia Y. Tretyakova,[b],* and Ashis K. Basu[a]

[a] Department of Chemistry, University of Connecticut, Storrs, Connecticut, USA.

[b] Department of Medicinal Chemistry and the Masonic Cancer Center, University of Minnesota, Minneapolis, Minnesota, USA. E-mail: trety001@umn.edu

[c] Department of Medicinal Chemistry and the Masonic Cancer Center, University of Minnesota, Minneapolis, Minnesota, USA. Current address: Department of Biotechnology, Alliance University, Anekal, Bengaluru - 562 106, Karnataka, India.

TABLE OF CONTENTS

EXPERIMENTAL SECTION 2

MATERIALS 2
SYNTHESIS OF PROTECTED-AMINOOXY LYSINE (OXY-LYS) 2
SYNTHESIS OF BENZYL (R)-2-(((BENZYLOXY)CARBONYL)AMINO)-5-HYDROXYPENTANOATE (2) 2
SYNTHESIS OF BENZYL (R)-2-(((BENZYLOXY)CARBONYL)AMINO)-5-((METHYLSULFONYL)OXY)PENTANOATE (3) 3
SYNTHESIS OF BENZYL (R)-2-(((BENZYLOXY)CARBONYL)AMINO)-5-((1,3-DIOXOISOINDOLIN-2YL) OXY)PENTANOATE (3A) 3
SYNTHESIS OF BENZYL(R)-5-(AMINOOXY)-2-(((BENZYLOXY)CARBONYL)AMINO) PENTANOATE (4) 3
SYNTHESIS OF BENZYL (R)-2-(BENZYLOXY)CARBONYL)AMINO)-5(TER BUTOXYCARBONYL) (AMINO)OXY) PENTANOATE (4A) 4
SYNTHESIS OF (R)-2-(9H-FLUOREN-9-YL)METHOXY)CARBONYL)AMINO)-5-(TERT-BUTOXYCARBONYL) AMINO)OXY) PENTANOIC ACID (5) 4
SYNTHESIS OF 7-DEAZA-DG-CONTAINING DNA–PEPTIDE CONJUGATES VIA OXIME LIGATION 12
REAGENTS 14
CONSTRUCTION AND CHARACTERIZATION OF PMS2 VECTOR CONTAINING A SINGLE 7-DEAZA-DPC LESION 14
REPLICATION IN HUMAN EMBRYONIC KIDNEY (HEK 293T) AND TLS POLYMERASE-DEFICIENT CELLS 15
DETERMINATION OF LESION BYPASS EFFICIENCIES AND MUTATION ANALYSIS 15
STATISTICAL ANALYSIS 15

OLIGONUCLEOTIDE SEQUENCES 16

7 DEAZA DPC SEQUENCE 16
7 DEAZA DHP DG SEQUENCE 16
IC SEQUENCE 17

SUPPLEMENTARY TABLES 17

SUPLEMENTARY TABLES, SCHEMES, AND FIGURES

TABLE S 1: REPLICATION OF DPC-CONTAINING CONSTRUCT IN HEK 293T CELLS AND VARIOUS POLYMERASE KNOCKOUT HEK 293T CELLS. 17
TABLE S 2: REPLICATION OF 7 DEAZA (NO DPC) IN HEK 293T CELLS AND VARIOUS POLYMERASE KNOCKOUT HEK 293T CELLS. 22

SCHEME S 1: SYNTHESIS OF PROTECTED OXY-LYSINE. 2
SCHEME S 2: SYNTHESIS OF SITE-SPECIFICALLY MODIFIED DNA OLIGODEOXYNUCLEOTIDE CONTAINING 7-DEAZA-7-(2,3-DIHYDROPROP-1-YL)-DG). 12

FIGURE S 1: 1H-NMR SPECTRUM OF COMPOUND 4. 6
FIGURE S 2: 13C-NMR SPECTRUM OF COMPOUND 4. 7

FIGURE S 3: 1H-NMR SPECTRUM OF COMPOUND 4A. 8
FIGURE S 4: 13C-NMR SPECTRUM OF COMPOUND 4A. 9
FIGURE S 5: 1H-NMR SPECTRUM OF COMPOUND 5. 10
FIGURE S 6: 13C-NMR SPECTRUM OF COMPOUND 5. 11
FIGURE S 7: ESI+ MASS SPECTRUM AND DECONVOLUTED SPECTRUM OF DPC FORMED BETWEEN 7-DEAZA-7-(2,3-DIHYDROPROP-1-YL)-DG CONTAINING DNA AND OXY- LYS CONTAINING PEPTIDE. 13
FIGURE S 8:CLONAL EXPANSION AND MUTATION ANALYSIS VIA HYBRIDIZATION AND SEQUENCING. 16
FIGURE S 9: OLIGONUCLEOTIDE SEQUENCES OF THE TEMPLATE STRAND NEAR THE INSERTION SITE, ITS COMPLEMENTARY STRAND, AND THE PROBES USED FOR HYBRIDIZATION. 17

Experimental Section

Materials

Solvents and reagents used for synthesis and purification were purchased from Sigma-Aldrich (St. Louis, MO, USA) or Thermo Fisher Scientific (Waltham, MA, USA), unless otherwise specified. Nucleoside phosphoramidites, solid supports, and all other reagents required for the solid-phase synthesis of DNA were obtained from Glen Research (Sterling, VA, USA). Synthetic DNA oligonucleotide was synthesized by solid phase synthesis using an ABI 394 DNA synthesizer (Applied Biosystems, CA).

Synthesis of protected-aminooxy lysine (oxy-Lys)

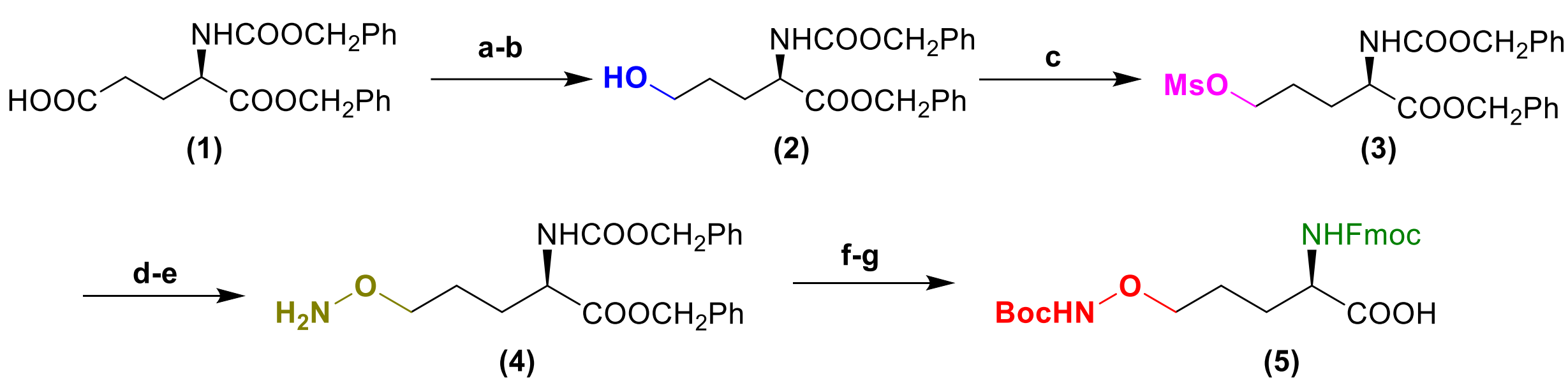

Scheme S 1: Synthesis of protected oxy-lysine. (a) Ethylchloroformate, *N*-methylmorpholine, THF, -10 °C; (b) $NaBH_4$, MeOH, 0 °C; (c) methanesulfonylchloride, Et_3N, DCM, °C; (d) *N*-hydroxypthalimide, DBU, DMF, 0 °C-RT. (e) methylhydrazine, DCM, 0 °C; (f) Boc_2O, Et_3N, THF, rt; (g) H_2/Pd/C, rt, FmocOsu, $NaHCO_3$, dioxane, H_2O, rt.

Synthesis of Benzyl (R)-2-(((benzyloxy)carbonyl)amino)-5-hydroxypentanoate (2)

This compound was synthesized as reported in literature.[1] The spectroscopic and analytical data were consistent with those reported in the literature. ^1H NMR ($CDCl_3$, 500 MHz): δ 7.26 (s, 10H), 5.40-5.41 (d,

1H, J = 6.5Hz), 5.06-5.13 (m, 2H), 5.03-5.5.05 (m, 2H), 4.37-4.41 (m, 1H), 3.54 (t, 1H, J = 6.1Hz), 1.84-1.1.91 (m, 1H), 1.67-1.74 (m, 1H), 1.42-1.55 (m, 1H). ^{13}C NMR ($CDCl_3$, 125 MHz): δ 172.1, 155.8, 140.9, 136.1135.2, 128.6, 128.5, 128.5, 128.3, 128.2, 128.1, 127.6, 126.967.4, 67.2, 67.1, 67.0, 65.3, 62.0, 60.4, 59.2, 53.6, 49.6, 29.3, 28.0, 25.5, 21.0, 20.9, 14.2.5

Synthesis of Benzyl (R)-2-(((benzyloxy)carbonyl)amino)-5-((methylsulfonyl)oxy)pentanoate (3)

This compound was synthesized as reported in literature.[2] The spectroscopic and analytical data were consistent with those reported in the literature. ^{1}H NMR ($CDCl_3$, 500 MHz): δ 7.28 (s, 10H), 5.27-5.29 (d, 1H, *J* = 7.5 Hz), 5.05-5.14 (m, 2H), 5.03 (s, 2H), 4.38-4.39 (d, 1H, *J* = 5Hz), 4.12 (s, 2H), 2.88 (s, 3H), 1.93-1.94 (d, 1H, *J* = 5.5Hz), 1.66-1.73 (m, 1H). ^{13}C NMR ($CDCl_3$, 125 MHz): δ 171.7, 155.9, 136.0, 135.0, 128.7, 128.6, 128.5, 128.4, 128.2, 128.1, 68.8, 67.4, 67.1, 60.4, 53.2, 50.8, 37.3, 28.9, 25.0, 21.0, 14.1

Synthesis of Benzyl (R)-2-(((benzyloxy)carbonyl)amino)-5-((1,3-dioxoisoindolin-2yl) oxy)pentanoate (3a)

This compound was synthesized as reported in literature.[2] The characterization data matches the reported data. ^{1}H NMR ($CDCl_3$, 500 MHz): δ 7.73-7.74 (m, 2H), 7.63-7.67 (m, 2H), 7.24-7.28 (m, 10H), 5.42-5.43 (d,*J* = 8 Hz, 1H), 5.12 (s, 2H), 5.02 (s, 2H), 4.39-4.43 (m, 1H), 4.11 (br s, 2H), 2.07-2.09 (m, 1H), 1.89-1.97 (m, 1H), 1.71-1.75 (m, 2H). ^{13}C NMR ($CDCl_3$, 125 MHz): δ 172.1, 163.7, 156.1136.4, 135.4, 134.6, 129.0, 128.7, 128.6, 128.5, 128.4, 128.2, 128.1, 123.6, 67.3, 67.1, 53.8, 28.9, 24.4.

Synthesis of Benzyl(R)-5-(aminooxy)-2-(((benzyloxy)carbonyl)amino) pentanoate (4)

To a stirred solution of compound **3a** (2.49 g, 4.9 mmol) in dichloromethane (60 mL) at 0 °C was added methylhydrazine (7.44 mmol, 392 µL). The reaction mixture was stirred at 0 °C for 1 h. Upon completion of the reaction, as monitored by TLC, the mixture was passed through a pad of Celite, and the filtrate was concentrated under reduced pressure. The resulting crude residue was purified by flash column chromatography to afford compound **3** (1.65 g, 89%) as a off-white solid. TLC (silica gel, CH2Cl2/MeOH, 90:10) *R*f 0.3. ESI+-MS: Exact mass calc. for $[C_{20}H_{23}N_2O_4]^+$ 355.1652 $[M + H]^+$, Exact mass obs: 355.1656 $[M + H]^+$. ^{1}H NMR ($CDCl_3$, 500 MHz): δ 7.35 (s, 11H), 5.44-5.46 (d, *J* = 8 Hz, 1H), 5.17-5.20 (m, 2H), 5.13 (s, 2H), 4.45-4.49 (m, 1H), 3.66-3.69 (m, 2H), 3.51 (s, 1H), 1.92-1.97 (m, 1H), 1.74- 1.86 (m, 1H), 1.60-1.69 (m, 1H). ^{13}C NMR ($CDCl_3$, 125 MHz): δ 172.2, 155.9, 136.2, 135.2, 128.6, 128.5, 128.5, 128.3, 128.2, 128.1, 74.9, 67.2, 67.0, 53.7, 50.8, 49.6, 24.0.

Synthesis of Benzyl (R)-2-(benzyloxy)carbonyl)amino)-5(ter butoxycarbonyl) (amino)oxy) pentanoate (4a)

To a solution of compound **4** (100 mg, 0.26 mmol) in dry THF (16 mL) was added triethylamine (54 mg, 0.52 mmol) and di-tert-butyl dicarbonate (116 mg, 0.52 mmol) and the reaction mixture was stirred at room temperature for 3hr. After completion of the reaction (as monitored by TLC), the mixture was evaporated to dryness under reduced pressure and subjected to flash column chromatography (FC) to give compound **4a** (90 mg, 72%) as a colourless oil. TLC (silica gel, EtOAc/Hexanes, 70:30) *R*f 0.5. ESI+-MS: Exact mass calc. for $[C_{25}H_{31}N_2O_6]^+$ 455.2177 $[M + H]^+$, Exact mass obs: 455.1215 $[M + H]^+$. ^{1}H NMR (CDCl3, 500 MHz): δ 7.23-7.27 (m, 11H), 7.11 (s, 1H), 5.49-5.51 (d, *J* = 8 Hz, 1H), 5.06-5.12 (m, 2H), 5.02 (s, 2H), 4.32-4.37 (m, 1H), 3.73 (t, 1H), 1.87-1.91 (m, 1H), 1.75-1.79 (m, 1H), 1.55-1.59 (m, 2H). ^{13}C NMR (CDCl3, 125 MHz): δ 172.2, 157.0, 156.1, 136.2, 135.3, 128.6, 128.5, 128.3, 128.1, 81.7, 75.8, 67.1, 66.9, 53.8, 29.0, 28.1, 23.9.

Synthesis of (R)-2-(9H-fluoren-9-yl)methoxy)carbonyl)amino)-5-(tert-butoxycarbonyl) amino)oxy) pentanoic acid (5)

Solution of compound **4a** (0.6 g, 1.27 mmol) in methanol (10 ml) at room temperature was stirred with 10% Pd/C (0.270 g, 2.53 mmol) under 1 atm H_2 for 2 hr. The Pd/C was removed by filteration and filtrate was concentrated. The residue was dissolved in dioxane/water mixture (1:1, v/v, 10 ml). To this solution was added 9-fluorenylmethylsuccinimidyl carbonate (FmocOsu) (0.51g, 1.51 mmol) and $NaHOC_3$ (0.23 g, 2.75 mmol) and stirred for overnight at room temperature. The reacion mixture was further acidified to pH 3-4 by addition of 1N HCl. After neutralization, the reaction solution was extracted with ethyl acetate (3x 30 ml) and the combined organic extracts were washed with water (30 ml) and brine (30 ml) and dried over anhydrous Na_2SO_4 and evaporated to dryness under reduced pressure. The residue was further purified by FC to obtain as viscous oil. Lyophilization from acetonitrile-water provided off-white solid (0.35 g, 59%). TLC (silica gel, DCM/ MeOH, 90:10) *R*f 0.3. ESI+-MS: Exact mass calc. for [C25H30N2NaO7]+ 493.1945 [M + Na]+, Exact mass obs: 493.1946 [M + Na]+. ^{1}H NMR (CDCl3, 500 MHz): δ 7.65 (bs, 2H), 7.50 (bs, 3H), 7.28 (bs, 2H), 7.12 (bs, 2H), 4.28-4.35 (m, 3H), 4.10 (bs, 1H), 3.76-3.78 (m, 2H), 1.94 (bs, 1H), 1.78

(bs, 1H), 1.62 (bs, 2H), 1.37 (s, 9H). ^{13}C NMR ($CDCl_3$, 125 MHz): δ 143.73, 141.25, 127.67, 127.09, 125.20, 119.92, 53.43, 47.10, 29.71, 28.22.

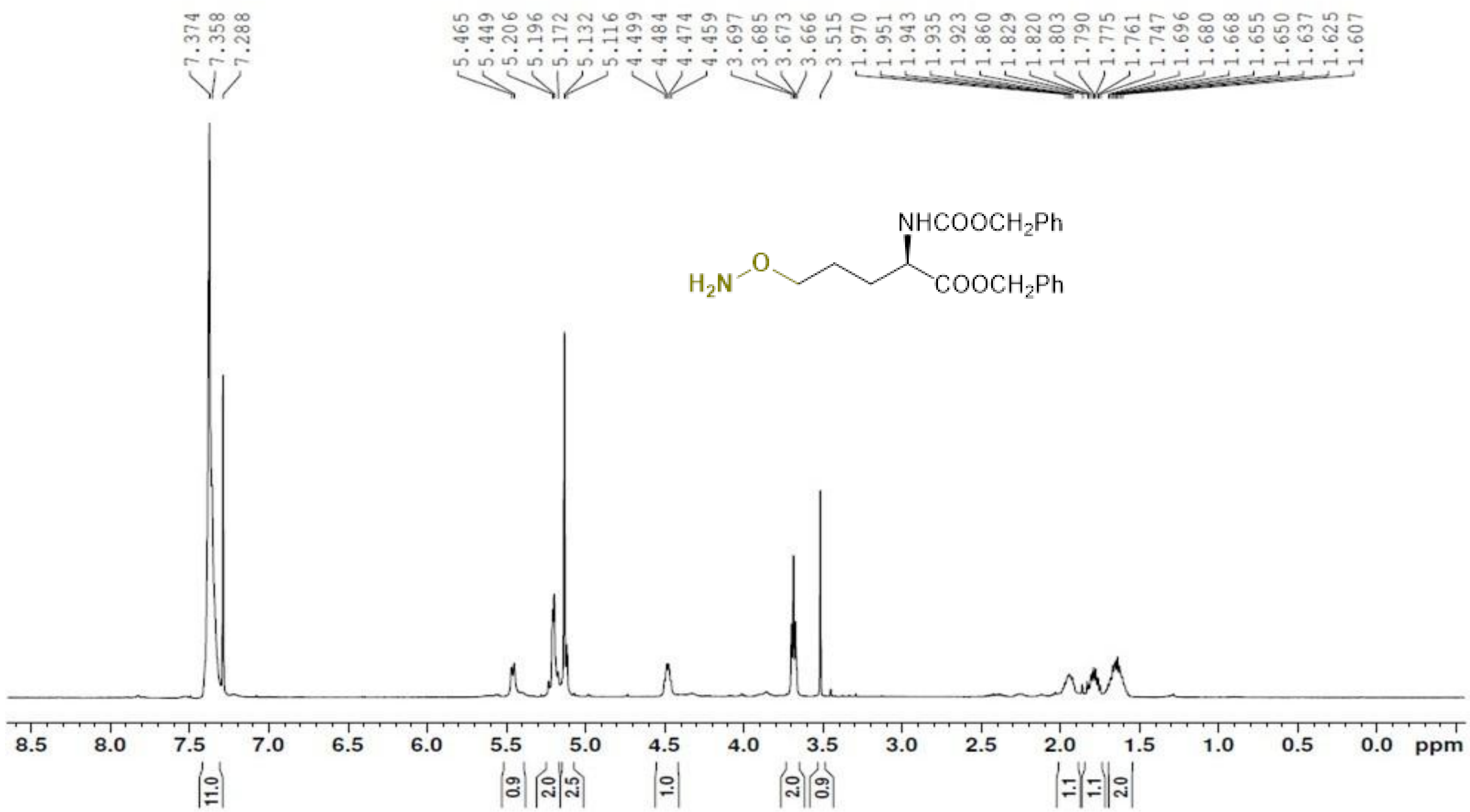

Figure S 1: 1H-NMR spectrum of compound 4.

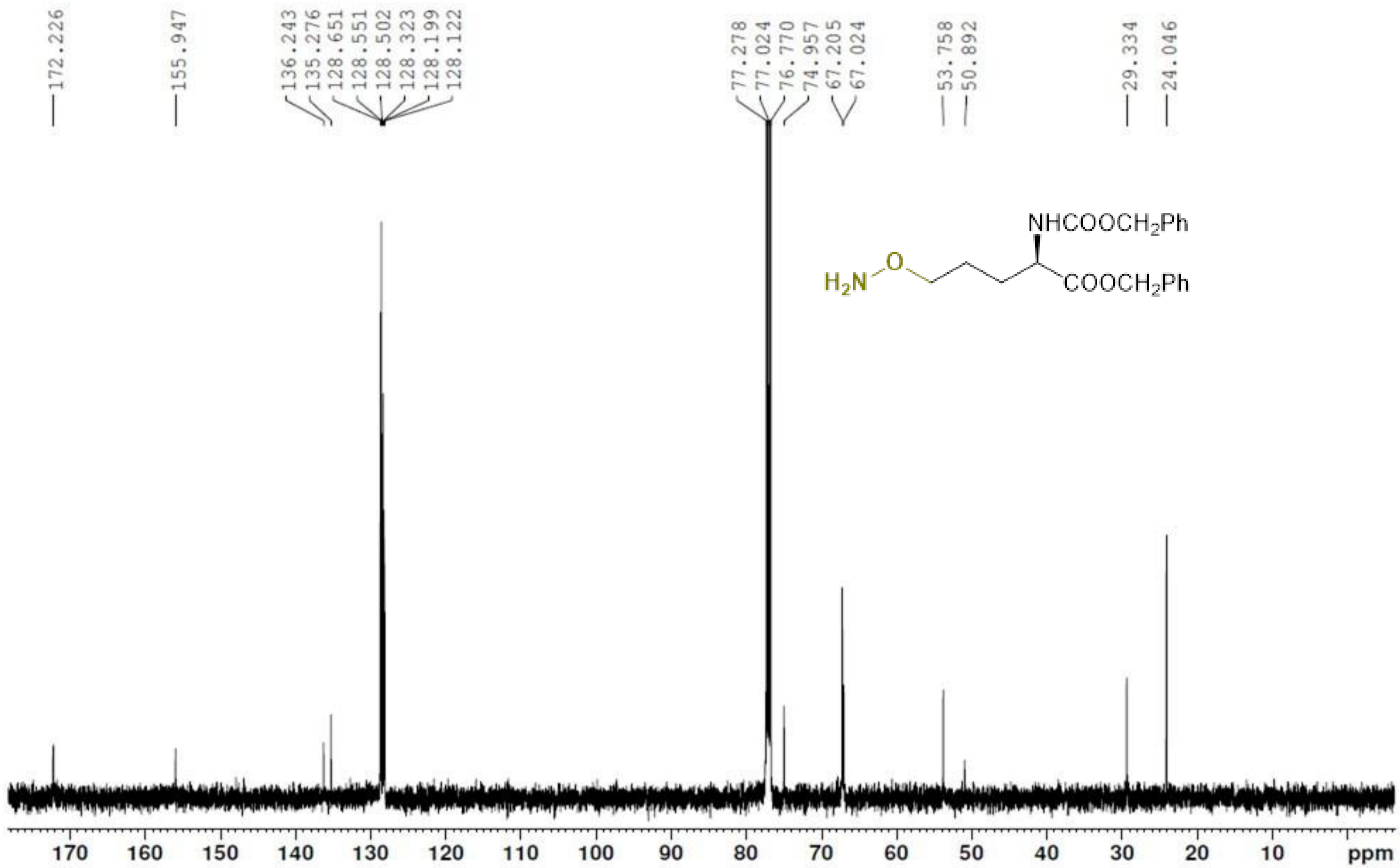

Figure S 2: 13C-NMR spectrum of compound 4.

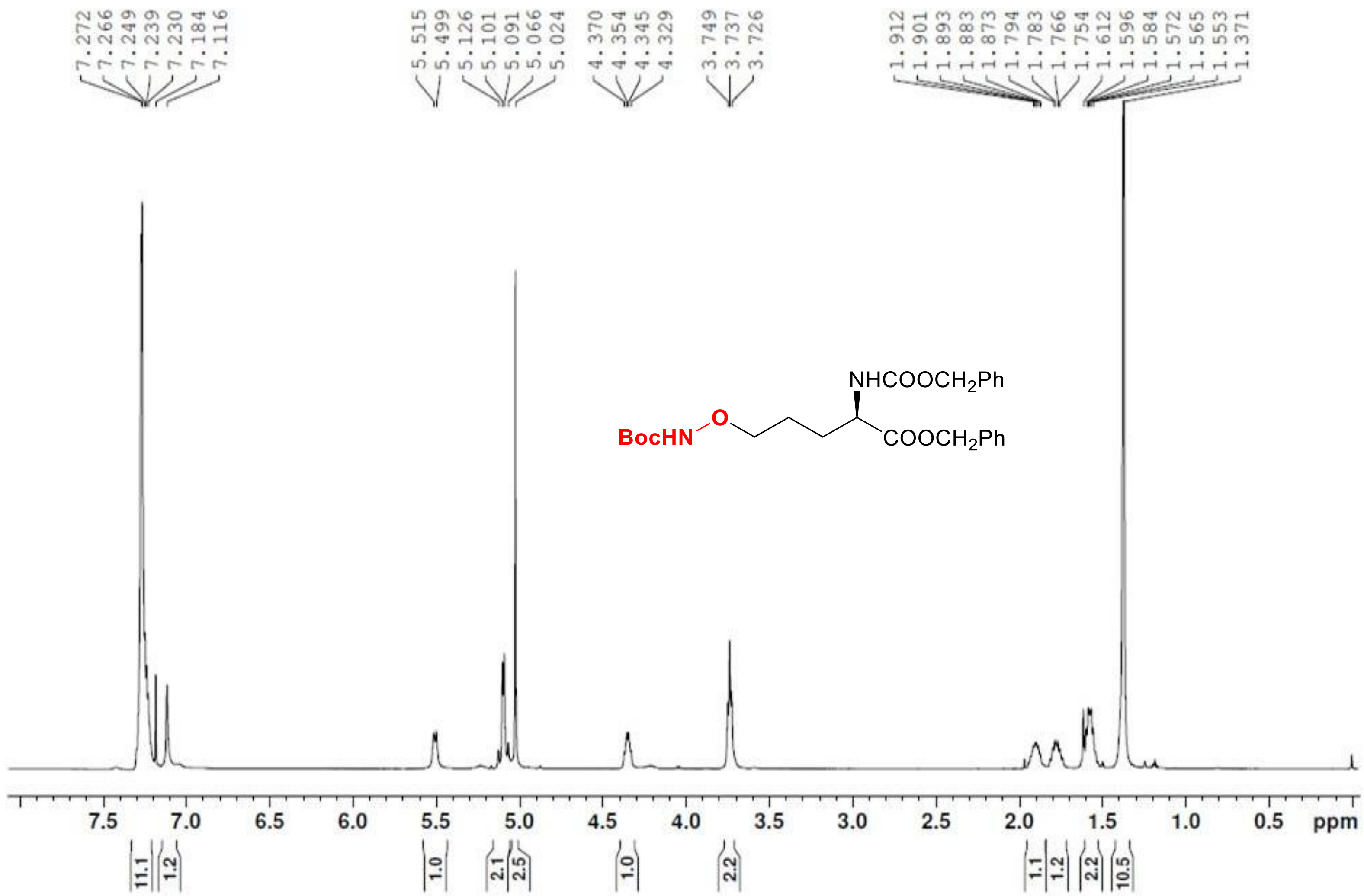

Figure S 3: 1H-NMR spectrum of compound 4a.

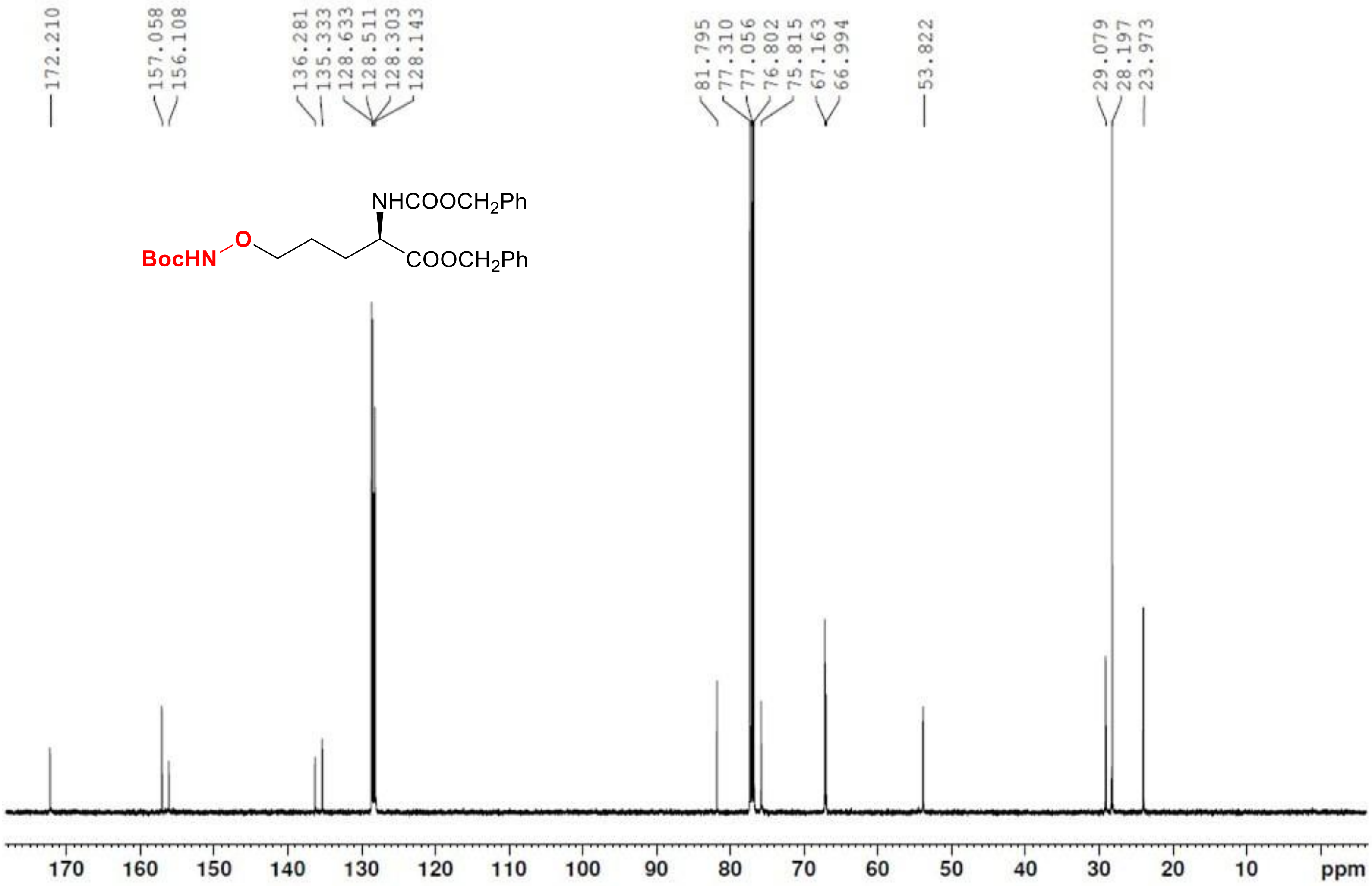

Figure S 4: 13C-NMR spectrum of compound 4a.

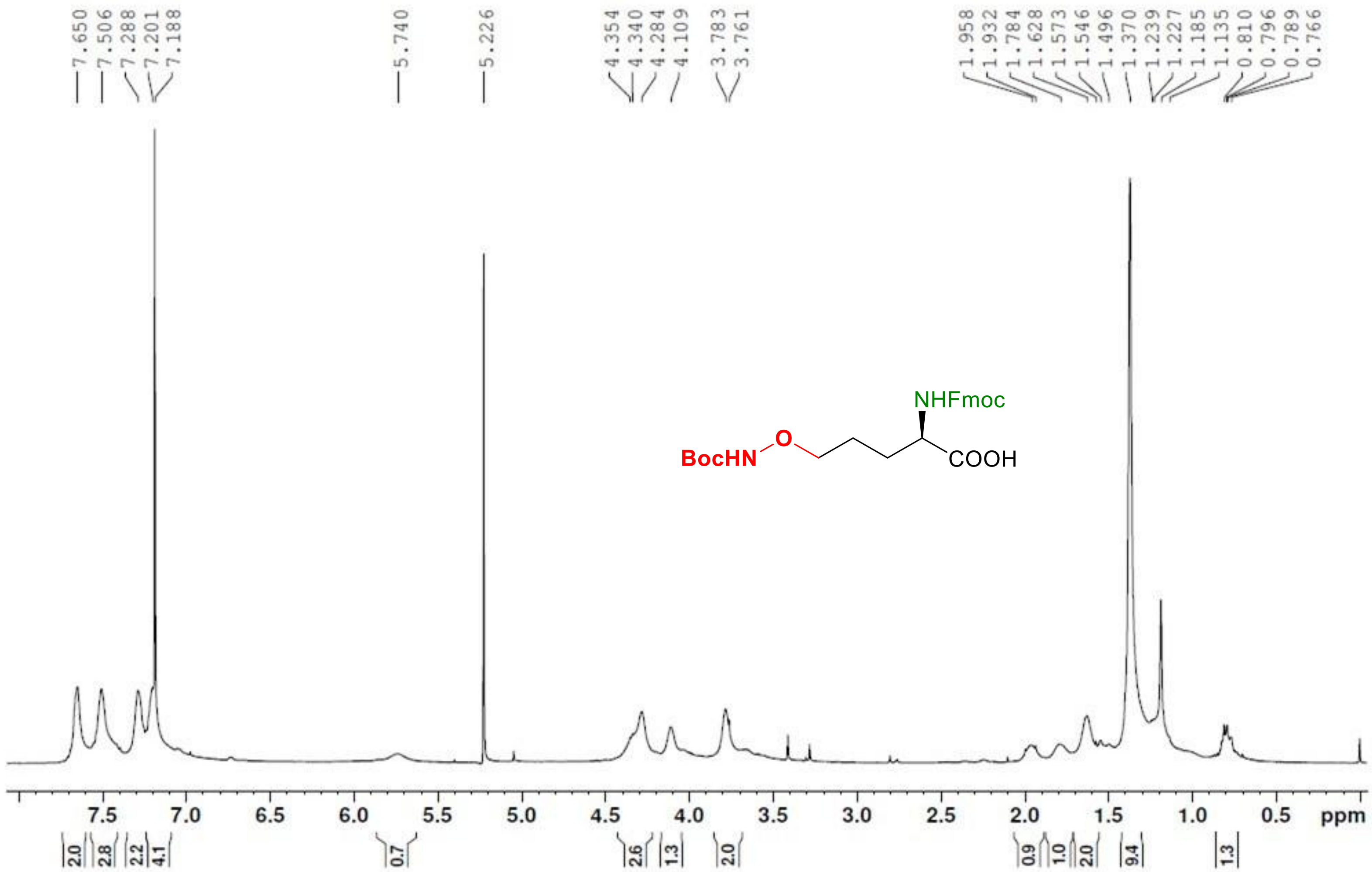

Figure S 5: 1H-NMR spectrum of compound 5.

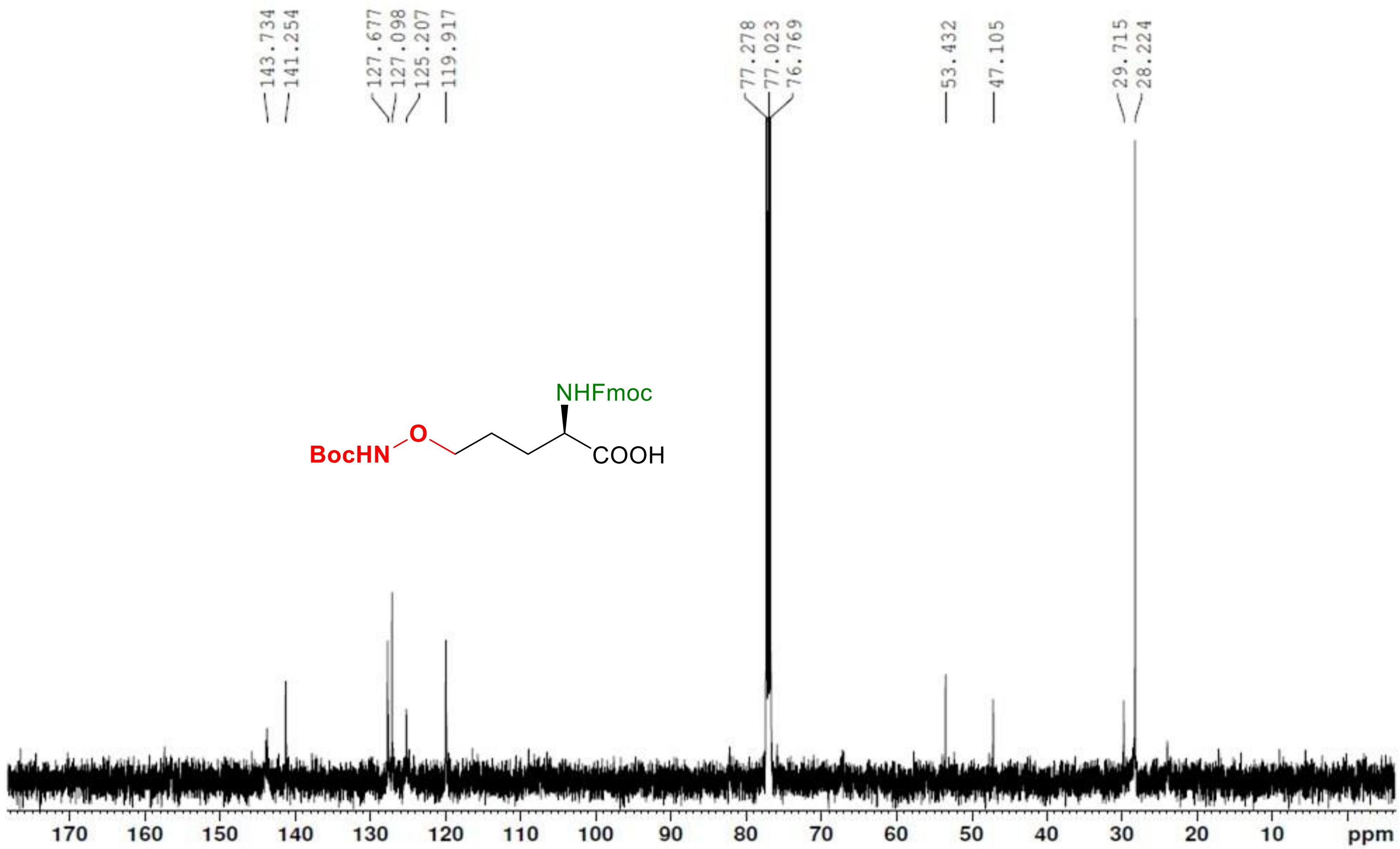

Figure S 6: 13C-NMR spectrum of compound 5.

Synthesis of 7-Deaza-dG-Containing DNA–Peptide Conjugates via Oxime Ligation

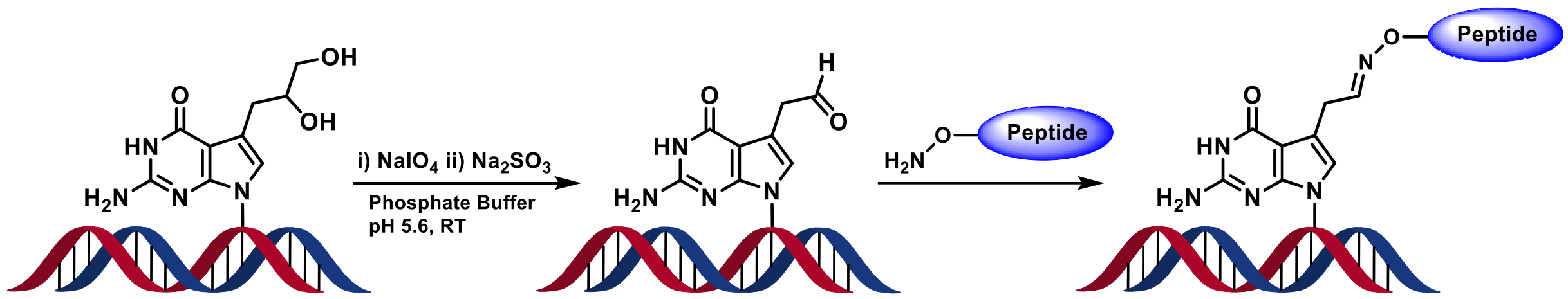

7-(2-oxoethyl)-7-deaza-dG

Scheme S 2: Synthesis of Site-specifically modified DNA oligodeoxynucleotide containing 7-deaza-7-(2,3-dihydroprop-1-yl)-dG).

Step 1. Oxidation of the DNA Aldehyde Precursor

To a solution of Site-specifically modified DNA oligodeoxynucleotide (ODN) containing 7-deaza-7-(2,3-dihydroprop-1-yl)-dG (200 pmol) in a microcentrifuge tube added sodium phosphate buffer (15 mM, pH 5.6; 10 µL of a 30 mM stock solution) and nuclease-free water to a final volume of 20 µL. Sodium periodate ($NaIO_4$, 10 mM final concentration; 10 µL of a 200 mM stock solution) was then added, and the reaction mixture was incubated for 6 h at 4 °C in the dark. Upon completion, the reaction was quenched by the addition of sodium sulfite (Na_2SO_3, 100 mM final concentration; 5 µL of a 500 mM stock solution). The reaction mixture was desalted using a NAP-5 column, and the oxidized DNA was concentrated under reduced pressure using a Speed-Vac to get approximately 20–25 pmol of DNA Aldehyde Precursor with 83% yield.

Step 2. Oxime Ligation with Peptide

The obtained DNA aldehyde precursor was treated with Sodium phosphate buffer (15 mM final concentration, pH 5.6; 10 µL of a 30 mM stock solution) was added to the residue, followed by the peptide (20 nmol, 100 equiv relative to DNA). Nuclease-free water was then added to adjust the total reaction volume to 20 µL. The reaction mixture was thoroughly mixed and incubated overnight at 37 °C to allow oxime bond formation between the aldehyde-functionalized DNA and the aminooxy-containing peptide. After completion, the reaction mixture was desalted on a NAP-5 column, concentrated using a Speed-Vac, and the resulting DNA–peptide conjugate (DPC, 52% yield) was characterized by LC–MS.

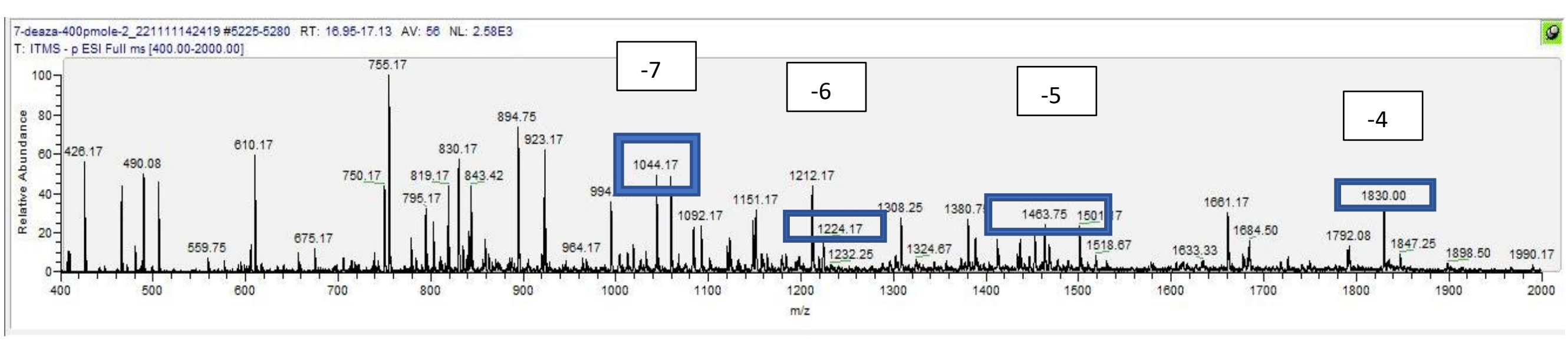

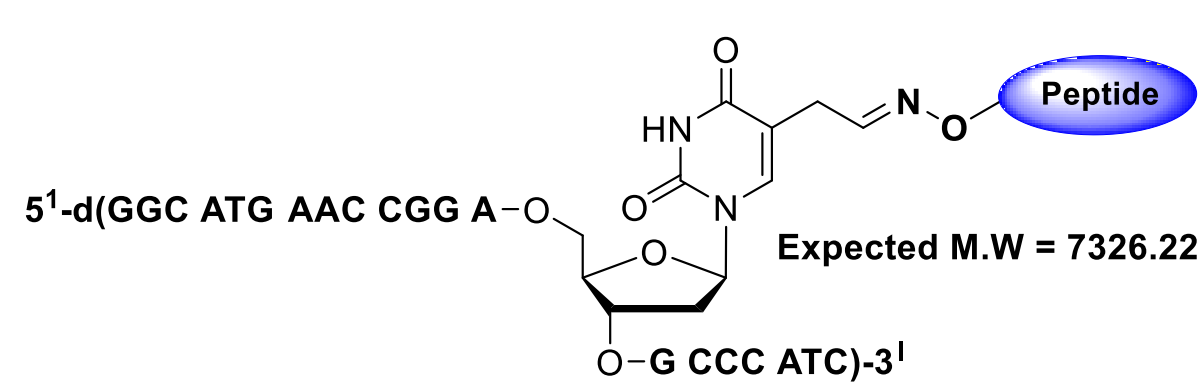

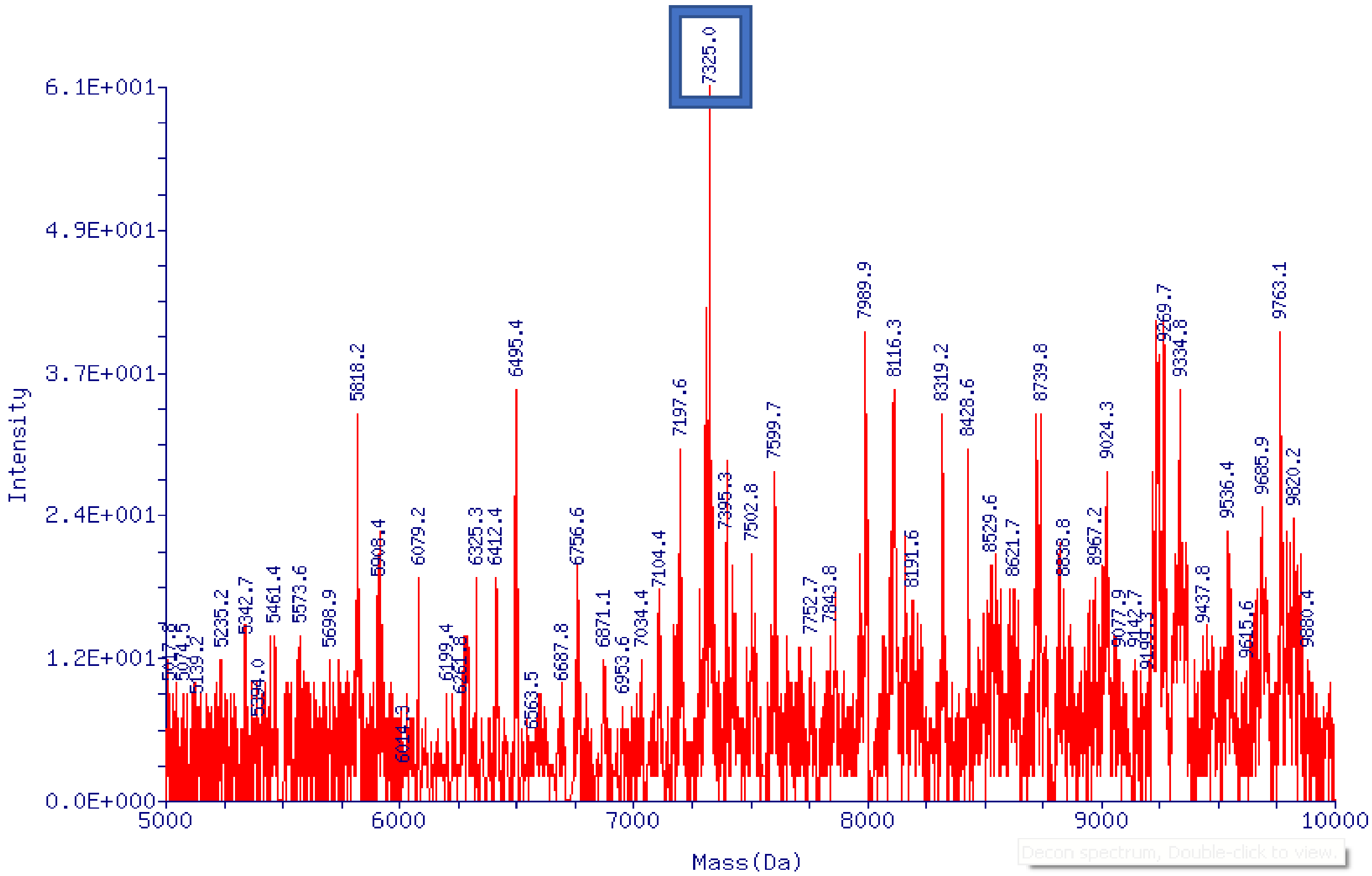

Figure S 7: ESI+ mass spectrum and deconvoluted spectrum of DPC formed between 7-deaza-7-(2,3-dihydroprop-1-yl)-dG containing DNA and oxy- Lys containing Peptide.

Purification

DNA samples were mixed with SDS loading dye and subjected to electrophoresis on a 10% SDS-polyacrylamide gel to separate the desired DNA substrate from reaction byproducts. The DNA band was excised, recovered from the gel, and purified. An aliquot of the purified DNA was then end-labeled with [γ-^{32}P] ATP and analyzed on a second 10% SDS-polyacrylamide gel. Following electrophoresis, the gel was dried and exposed to a phosphor screen, and radiolabeled DNA was visualized by phosphor imaging to assess sample purity.

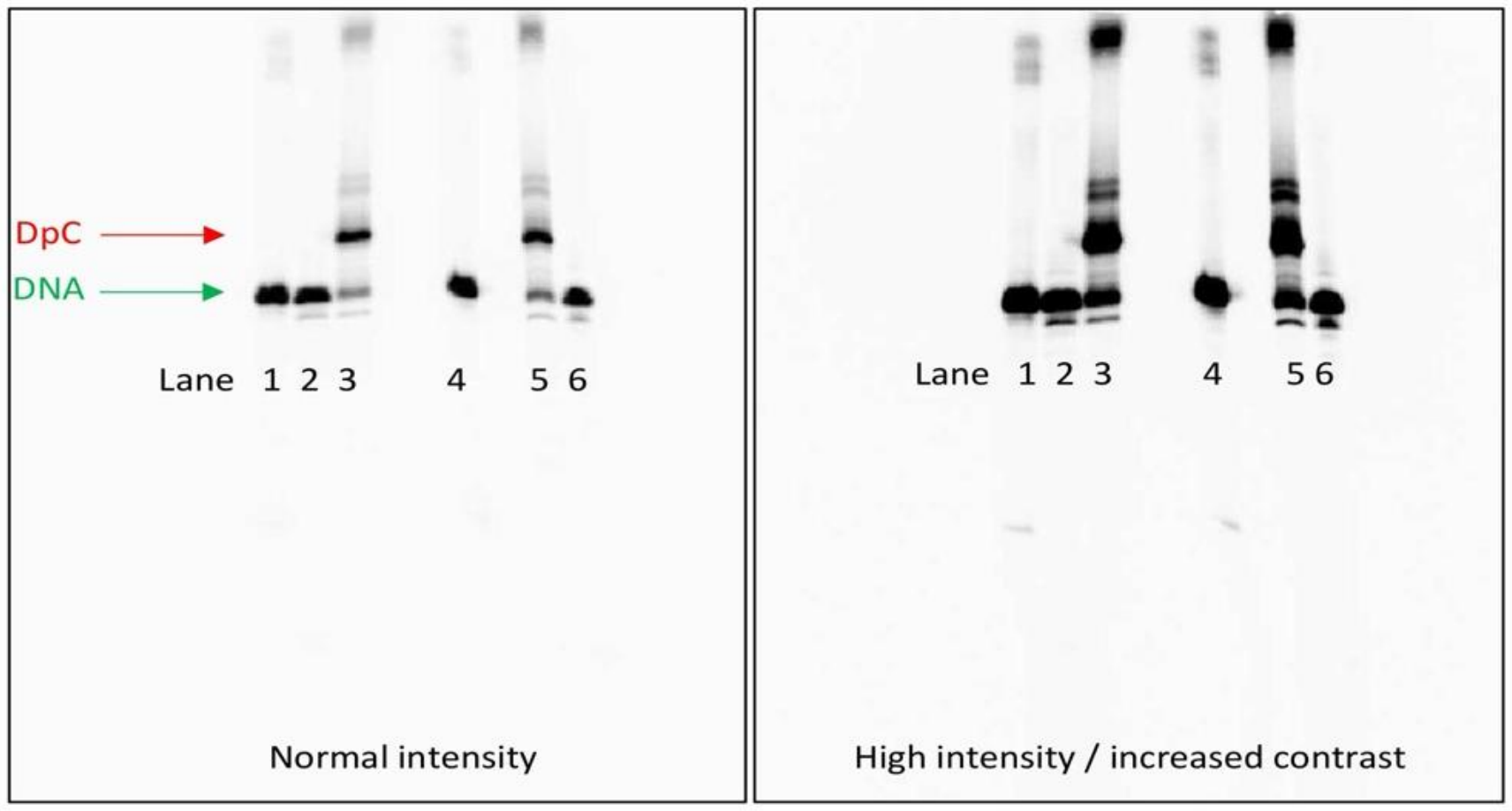

Lane	Sample
1	Control: codon 248
2	Control: codon 249
3	DNA lesion: 7-deaza (codon 249)
4	Control: codon 248
5	DNA lesion: 7-deaza (codon 249)
6	Control: codon 249

5'-GGC ATG AAC CG*G AGG CCC ATC-3'
Codon 248

5'- GGC ATG AAC CGG AG*G CCC ATC-3'
Codon 249

Figure S 8: Purification of DpC

Reagents

All chemicals and solvents were of analytical grade and used without further purification unless otherwise stated. Restriction endonuclease EcoRV-HF, T4 DNA ligase, T4 polynucleotide kinase, uracil DNA glycosylase, and exonuclease III were purchased from New England Biolabs. The lesion-containing oligodeoxynucleotide used in this study had the sequence 5′–GGCATGAACCGGAG*GCCCATC–3′, where G* denotes 7-deaza-DHP-dG covalently linked to the lysine residue (K*) of an 11-mer peptide (NH_2–GGGKGLGKGGA). Control oligodeoxynucleotides contained the same DNA sequence with G replaced by unmodified 7-deaza-DHP-dG. All unmodified oligonucleotides, including scaffolds and probes, were purchased from Integrated DNA Technologies, and their sequences are provided in the supplementary data. [γ-^{32}P] ATP was obtained from Revvity Inc. (formerly PerkinElmer Health Sciences). Plasmid pMS2 was kindly provided by Professor M. Moriya (Stony Brook University, The State University of New York). Human embryonic kidney (HEK 293T) cells were obtained from the American Type Culture Collection (Manassas, VA) (ATCC Cat # CRL-3216, RRID:CVCL_0063). TLS polymerase-deficient HEK 293T cell lines (lacking hPol η, hPol κ, hPol ι, hPol ζ) were generously provided by Professor Yinsheng Wang (University of California, Riverside) and generated via CRISPR-Cas9 genome editing[4,5].

Construction and Characterization of pMS2 Vector Containing a Single 7-Deaza-DpC Lesion

The single lesion pMS2 vector, harboring ampicillin and neomycin resistance markers, was constructed using a site-specific ligation strategy. Briefly, 5 µg of pMS2 DNA was digested with excess EcoRV-HF at 37 °C for 5 h. The digested plasmid was annealed with a 56-mer oligonucleotide scaffold containing four

uracil bases by heating to 75 °C followed by slow cooling to room temperature and overnight incubation at 4 °C to generate a gapped circular DNA intermediate. Control and lesion-containing oligonucleotides were phosphorylated with T4 polynucleotide kinase, annealed to the gapped vector, and ligated overnight at 16 °C. For the DpC construct, HpaII enzyme was added to remove control strand (C/CGG cut site). The scaffold strand was subsequently removed by digestion with uracil DNA glycosylase and exonuclease III at 16 °C for 13 h. Unligated oligonucleotides and residual salts were eliminated using Amicon Ultra-0.5 mL centrifugal filters (100K MWCO, Millipore Sigma). Circularization efficiency was assessed by electrophoresis on a 1% agarose gel, confirming ≥40% ligation efficiency across the gap.

Replication in Human Embryonic Kidney (HEK 293T) and TLS Polymerase-Deficient Cells

HEK 293T cells were cultured in Dulbecco's Modified Eagle's Medium supplemented with 4 mM L-glutamine, 1.5 g/L sodium bicarbonate, 4.5 g/L glucose, and 10% fetal bovine serum. Cells were maintained at 37 °C in a humidified 5% CO_2 atmosphere and used for transfection at 70–80% confluence. For each transfection, 60 ng of lesion-containing pMS2 and 60 ng of an internal control (IC) vector, harboring a distinct 12-mer sequence at the ligation site, were combined with Lipofectamine (3.6% final concentration; Invitrogen) in Opti-MEM medium (Gibco). For controls, unmodified pMS2 vectors containing 7-deaza-DHP-dG at the corresponding site were co-transfected with the IC construct at a 1:1 molar ratio. After 15 min incubation at room temperature to allow liposome complex formation, the mixtures were added to the cells. Following 20–22 h incubation at 37 °C, cells were harvested using RLT lysis buffer (Qiagen) supplemented with β-mercaptoethanol (Sigma). The same experimental procedure was applied to TLS polymerase-deficient cell lines.

Determination of Lesion Bypass Efficiencies and Mutation Analysis

Progeny plasmids were recovered using the QiaPrep Spin Miniprep Kit (QIAGEN), yielding typically 90–120 ng DNA. The recovered DNA was amplified by transformation into E. coli DH10B, and transformants were analyzed using oligonucleotide hybridization. Probes complementary to the insertion site (central probe) and to sequences flanking the ligation site (left and right probes) were used to distinguish correctly replicated sequences from mutants.

Colonies hybridizing with all three probes were scored as faithful bypass events, while those hybridizing only with the flanking probes and not with the central probe were considered putative mutants and subjected to DNA sequencing. Colonies failing to hybridize with the flanking probes were excluded from analysis. TLS efficiency was calculated as the ratio of colonies derived from lesion-containing plasmids to those from the internal control vector.

Statistical Analysis

Data are presented as mean ± standard deviation (SD). The sample size (*n*) for each analysis is indicated in each figure legend..p-values are derived from two-tailed, unpaired Student's t-test. All statistical tests were performed using GraphPad Prism software (v8).

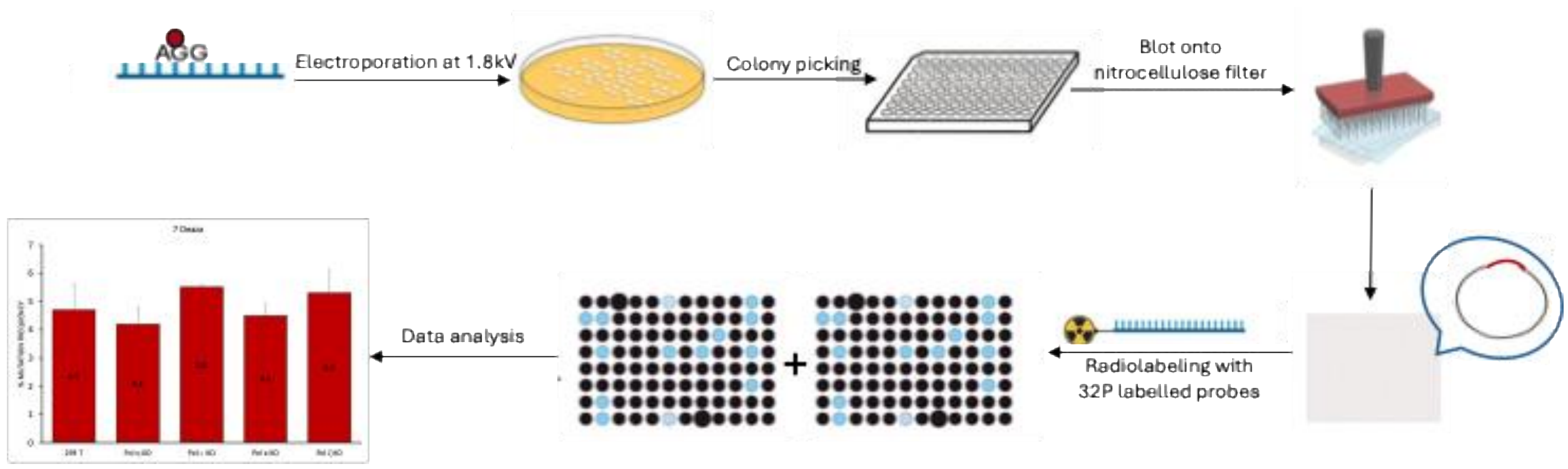

Figure S 9:Clonal Expansion and Mutation Analysis via Hybridization and Sequencing.

Oligonucleotide Sequences

7 Deaza DpC sequence

Scaffold
3'-CTC GAC CCA UGG UCG CTA CCG TAC TTG GCC TCC GGG TAG UAG CGA ACG UCC CCG GGA GCT-5'
Template strand
5'-…GGT ACC AGC GAT GGC ATG AAC CGG AG*G CCC ATC ATC GCT TGC AGG…-3'
Complementary strand
3'-…CCA TGG TCG CTA CCG TAC TTG GCC TCC GGG TAG TAG CGA ACG TCC…-5'
5'- CCC ATC ATC GCT TGC-3' (Right probe)
5'-GAT GGC ATG AAC CG-3'
(Central probe)

Peptide: NH_2-GGG KGL GK*G GA-CO2H
G* = 7-deaza-dG, K* = Derived from the N-terminal region of histone H4, where Lys-8 was replaced with oxy-Lys

7 Deaza DHP dG sequence

Scaffold
3'-CTC GAC CCA UGG UCG CTA CCG TAC TTG GCC TCC GGG TAG UAG CGA ACG UCC CCG GGA GCT-5'
Template strand
5'-…GGT ACC AGC GAT GGC ATG AAC CGG AG*G CCC ATC ATC GCT TGC AGG…-3'
Complementary strand
3'-…CCA TGG TCG CTA CCG TAC TTG GCC TCC GGG TAG TAG CGA ACG TCC…-5'
5'- CCC ATC ATC GCT TGC-3' (Right probe)
5'-GAT GGC ATG AAC CG-3'
(Central probe)

IC sequence

Scaffold

3'-CTC GAC CCA UGG UCG CTA AAG GGC CAG TGC TGC AA UAG CGA ACG UCC CCG GGA GCT-5'

Template strand

5'-…GGT ACC AGC GAT TTC CXG GTC ACG ACG TT ATC GCT TGC AGG…-3'

Complementary strand

3'-…CCA TGG TCG CTA AAG GGC CAG TGC TGC AA TAG CGA ACG TCC…-5'

5'-G ACG TT ATC GCT TGC-3' (Right probe)

5'-GAT TTC CCG GTC AC -3'
(Central probe)

Figure S 10: Oligonucleotide sequences of the template strand near the insertion site, its complementary strand, and the probes used for hybridization. **Underlined bases are digested site by EcorV-HF. In red are the inserted bases in the complementary strand and the uracil bases in the scaffold.**

Supplementary Tables

Table S 1: Replication of DpC-containing construct in HEK 293T cells and various polymerase knockout HEK 293T cells.

7-deaza DpC (Codon 249): %TLS EFFICIENCY							
**Sample**	**Trial 1**	**Trial 2**	**Trial 3**	**Trial 4**	**Average**	**SD**	**p-values**
293T WT	83	79	86		82.67	3.51	
Pol η KO	60	57			58.50	2.12	0.00243052
Pol ι KO	51	53	54		52.67	1.53	0.00138392
Pol κ KO	66	60			63.00	4.24	0.0351757
Pol ζ KO	73	79			76.00	4.24	0.21206869

7-deaza DpC: Codon 249							
**SAMPLE**	**DATA**	**Trial 1**	**Trial 2**	**Trial 3**	**Trial 4**	**Ave**	**SD**
**7D DpC (249) 293T**	# of mutants	29	22	25	17.0	9.4	1.0
	# of WT	248	248	240	161.0		
	Total # of colonies	277	270	265	178		
	%MF	10.5	8.1	9.4	9.6		
	**DATA**	**Trial 1**	**Trial 2**	**Trial 3**	**Trial 4**	**Ave**	**SD**
	G* → A	10	9	7		8.7	1.5
	G* → T	4	3	4		3.7	0.6
	G* → C	0	0	0		0.0	0.0
	G* del	0	0	0		0.0	0.0
	Off-target	5	2	5		4.0	1.7
	Double mutations	10	7	8		8.3	1.5

	Triple mutations	0	1	1		0.7	0.6
	**% MUTATION FREQUENCY**						
	G* → A	3.6	3.3	2.6		3.2	0.5
	G* → T	1.4	1.1	1.5		1.4	0.2
	G* → C	0.0	0.0	0.0		0.0	0.0
	G* del	0.0	0.0	0.0		0.0	0.0
	Off-target	1.8	0.7	1.9		1.5	0.6
	Double mutation	3.6	2.6	3.0		3.1	0.5
	Triple mutations	0.0	0.4	0.4		0.2	0.2
	Total %MF	10.5	8.1	9.4		9.4	1.2

**7-deaza DpC: Codon 249**							
**SAMPLE**	**DATA**	**Trial 1**	**Trial 2**	**Trial 3**	**Trial 4**	**Ave**	**SD**
**7D DpC (249) HKO**	# of mutants	38	43	39	39.0	18.5	0.4
	# of WT	193	187	162	163.0		
	Total # of colonies	231	230	201	202		
	%MF	16.5	18.7	19.4	19.3		
	**DATA**	**Trial 1**	**Trial 2**	**Trial 3**	**Trial 4**	**Ave**	**SD**
	G* → A	11	13	12		12.0	1.0
	G* → T	10	11	9		10.0	1.0
	G* → C	0	0	0		0.0	0.0
	G* del	0	0	0		0.0	0.0
	Off-target	9	9	9		9.0	0.0
	Double mutations	8	10	9		9.0	1.0
	Triple mutations	0	0	0		0.0	0.0
	**% MUTATION FREQUENCY**						
	G* → A	4.8	5.7	6.0		5.5	0.6
	G* → T	4.3	4.8	4.5		4.5	0.2
	G* → C	0.0	0.0	0.0		0.0	0.0
	G* del	0.0	0.0	0.0		0.0	0.0
	Off-target	3.9	3.9	4.5		4.1	0.3
	Double mutation	3.5	4.3	4.5		4.1	0.6
	Triple mutations	0.0	0.0	0.0		0.0	0.0
	Total %MF	16.5	18.7	19.4		18.2	1.5

**7-deaza DpC: Codon 249**							
**SAMPLE**	**DATA**	**Trial 1**	**Trial 2**	**Trial 3**	**Trial 4**	**Ave**	**SD**

7D DpC (249) KKO	# of mutants	23	25	20	20.0	10.0	0.6
	# of WT	199	220	197	172.0		
	Total # of colonies	222	245	217	192		
	%MF	10.4	10.2	9.2	10.4		
	**DATA**	**Trial 1**	**Trial 2**	**Trial 3**	**Trial 4**	**Ave**	**SD**
	G* → A	6	6	4		5.3	1.2
	G* → T	6	6	5		5.7	0.6
	G* → C	0	2	0		0.7	1.2
	G* del	0	0	0		0.0	0.0
	Off-target	7	7	7		7.0	0.0
	Double mutations	4	3	4		3.7	0.6
	Triple mutations	0	1	0		0.3	0.6
	**% MUTATION FREQUENCY**						
	G* → A	2.7	2.4	1.8		2.3	0.4
	G* → T	2.7	2.4	2.3		2.5	0.2
	G* → C	0.0	0.8	0.0		0.3	0.5
	G* del	0.0	0.0	0.0		0.0	0.0
	Off-target	3.2	2.9	3.2		3.1	0.2
	Double mutation	1.8	1.2	1.8		1.6	0.3
	Triple mutations	0.0	0.4	0.0		0.1	0.2
	Total %MF	10.4	10.2	9.2		9.9	0.6

**7-deaza DpC: Codon 249**							
**SAMPLE**	**DATA**	**Trial 1**	**Trial 2**	**Trial 3**	**Trial 4**	**Ave**	**SD**
7D DpC (249) IKO	# of mutants	14	15	7		13.9	2.4
	# of WT	184	108	38			
	Total # of colonies	198	123	45			
	%MF	7.1	12.2	15.6			
	**DATA**	**Trial 1**	**Trial 2**	**Trial 3**	**Trial 4**	**Ave**	**SD**
	G* → A	5	6	3		4.7	1.5
	G* → T	6	4	2		4.0	2.0
	G* → C	0	0	0		0.0	0.0
	G* del	0	0	0		0.0	0.0
	Off-target	1	1	1		1.0	0.0

	Double mutations	1	2	1		1.3	0.6
	Triple mutations	1	2	0		1.0	1.0
	**% MUTATION FREQUENCY**						
	G* → A	2.5	4.9	6.7		4.7	2.1
	G* → T	3.0	3.3	4.4		3.6	0.8
	G* → C	0.0	0.0	0.0		0.0	0.0
	G* del	0.0	0.0	0.0		0.0	0.0
	Off-target	0.5	0.8	2.2		1.2	0.9
	Double mutation	0.5	1.6	2.2		1.5	0.9
	Triple mutations	0.5	1.6	0.0		0.7	0.8
	Total %MF	7.1	12.2	15.6		11.6	4.3

**7-deaza DpC: Codon 249**							
**SAMPLE**	**DATA**	**Trial 1**	**Trial 2**	**Trial 3**	**Trial 4**	**Ave**	**SD**
	# of mutants	12	9	11		6.0	0.7
	# of WT	184	163	155			
	Total # of colonies	196	172	166			
	%MF	6.1	5.2	6.6			
	**DATA**	**Trial 1**	**Trial 2**	**Trial 3**	**Trial 4**	**Ave**	**SD**
	G* → A	4	3	4		3.7	0.6
	G* → T	3	3	3		3.0	0.0
	G* → C	0	2	0		0.7	1.2
	G* del	0	0	0		0.0	0.0
	Off-target	5	1	4		3.3	2.1
**7D DpC (249) ZKO**	Double mutations	0	0	0		0.0	0.0
	Triple mutations	0	0	0		0.0	0.0
	**% MUTATION FREQUENCY**						
	G* → A	2.0	1.7	2.4		2.1	0.3
	G* → T	1.5	1.7	1.8		1.7	0.1
	G* → C	0.0	1.2	0.0		0.4	0.7
	G* del	0.0	0.0	0.0		0.0	0.0
	Off-target	2.6	0.6	2.4		1.8	1.1
	Double mutation	0.0	0.0	0.0		0.0	0.0
	Triple mutations	0.0	0.0	0.0		0.0	0.0
	Total %MF	6.1	5.2	6.6		6.0	0.7

Double mutations			
**293T WT**	Trial 1	Trial 2	Trial 3
G7 → T + G6* del	2		1
G7 → T + G6* → A	3		1
G6* → T + G4 → T	3	1	2
G6* → T + G5 → T	1	1	
G6* del + A4 → T	1	1	
G6* → A + G4 → T		2	2
G6* → A + A4 → T		2	
G7 → T + G6* → T			2

Triple mutations			
**293T WT**	Trial 1	Trial 2	Trial 3
G6* → T + A4 → C + G5 → A		1	
G7 → T + G6* → A + A4 → G			1

Double mutations			
**HKO**	Trial 1	Trial 2	Trial 3
G6 → T + G4→T	3		1
G6 → T + G7→T	2	2	
G6* → A + G4 → A	1	2	2
G6* → A + G4 → T	1	1	
G6* → A + G5 → A		1	
G6 → T + G5→T		1	2
G6 → A + G7→T		1	1
G6 → A + G7→C		1	
G5 → A + G6* → T			1
G6 → T + C5 → T		1	1
C3 → T + G6 → T	1		1

Double mutations			
**KKO**	Trial 1	Trial 2	Trial 3
G6 → T + G4→C	1	1	1
G6 → T + G7→T	1		1
C3 → T + G6 → T		1	
G6* → A + G4 → T	1		
G6* → A + G5 → T		1	1
G6* → A + A4 → T	1		1

Triple mutations

KKO	Trial 1	Trial 2	Trial 3
C4 → T + C5 → T + G6*→T		1	

Double mutations			
IKO	Trial 1	Trial 2	Trial 3
G6 → T + G7→T			1
G6* → A + G4 → T	1	2	

Triple mutations			
IKO	Trial 1	Trial 2	Trial 3
G6 → T + G5 → A + G4→T	1	1	
G6 → A + C5 → A + G4→T		1	

Table S 2: Replication of 7 deaza (no DpC) in HEK 293T cells and various polymerase knockout HEK 293T cells.

7-deaza DHP dG: %TLS EFFICIENCY						
**Sample**	**Trial 1**	**Trial 2**	**Trial 3**	**Average**	**SD**	**p-values**
293T WT	86	88	86	86.67	1.15	0.0155102
Pol η KO	83	82		82.50	0.71	
Pol ι KO	84	84	90	86.00	3.46	
Pol κ KO	85	84		84.50	0.71	0.08063905
Pol ζ KO	90	85		87.50	3.54	0.79624196

SAMPLE	DATA	Trial 1	Trial 2	Trial 3	Ave	SD
7-deaza 293T WT	# of mutants	8	6	9	4.7	0.9
	# of WT	157	155	154		
	Total # of colonies	165	161	163		
	%MF	4.8	3.7	5.5		
	**DATA**	**Trial 1**	**Trial 2**	**Trial 3**	**Ave**	**SD**
	G6* → A	0	0	0	0.0	0.0
	G6* → T	0	0	0	0.0	0.0
	G6* → C	0	0	1	0.3	0.6
	G6*/G7 del	8	6	8	7.3	1.2
	Off-target	0	0	0	0.0	0.0
	**% MUTATION FREQUENCY**					
	G6* → A	0.0	0.0	0.0	0.0	0.0
	G6* → T	0.0	0.0	0.0	0.0	0.0
	G6* → C	0.0	0.0	0.6	0.2	0.4
	G6*/G7 del	4.8	3.7	4.9	4.5	0.7
	Off-target	0.0	0.0	0.0	0.0	0.0
	Total %MF	4.8	3.7	5.5	4.7	0.9

SAMPLE	DATA	Trial 1	Trial 2	Trial 3	Ave	SD
**7-deaza 293T HKO**	# of mutants	9	10	11	4.2	0.6
	# of WT	236	232	214		
	Total # of colonies	245	242	225		
	%MF	3.7	4.1	4.9		
	**DATA**	**Trial 1**	**Trial 2**	**Trial 3**	**Ave**	**SD**
	G6* → A	1	1	1	1.0	0.0
	G6* → T	0	0	1	0.3	0.6
	G6* → C	0	0	1	0.3	0.6
	G6*/G7 del	8	9	8	8.3	0.6
	Off-target	0	0	0	0.0	0.0
	**% MUTATION FREQUENCY**					
	G6* → A	0.4	0.4	0.4	0.4	0.0
	G6* → T	0.0	0.0	0.4	0.1	0.3
	G6* → C	0.0	0.0	0.4	0.1	0.3
	G6*/G7 del	3.3	3.7	3.6	3.5	0.2
	Off-target	0.0	0.0	0.0	0.0	0.0
	Total %MF	3.7	4.1	4.9	4.2	0.6

SAMPLE	DATA	Trial 1	Trial 2	Trial 3	Ave	SD
**7-deaza 293T KKO**	# of mutants	13	13	14	5.5	0.1
	# of WT	225	218	239		
	Total # of colonies	238	231	253		
	%MF	5.5	5.6	5.5		
	**DATA**	**Trial 1**	**Trial 2**	**Trial 3**	**Ave**	**SD**
	G6* → A	0	0	1	0.3	0.6
	G6* → T	0	0	0	0.0	0.0
	G6* → C	1	0	1	0.7	0.6
	G6*/G7 del	12	13	11	12.0	1.0
	Off-target	0	0	1	0.3	0.6
	**% MUTATION FREQUENCY**					
	G6* → A	0.0	0.0	0.4	0.1	0.2
	G6* → T	0.0	0.0	0.0	0.0	0.0
	G6* → C	0.4	0.0	0.4	0.3	0.2
	G6*/G7 del	5.0	5.6	4.3	5.0	0.6
	Off-target	0.0	0.0	0.4	0.1	0.2
	Total %MF	5.5	5.6	5.5	5.5	0.1

SAMPLE	DATA	Trial 1	Trial 2	Trial 3	Ave	SD
**7-deaza 293T IKO**	# of mutants	4	6	5	4.5	0.4
	# of WT	90	115	109		

	DATA	Trial 1	Trial 2	Trial 3	Ave	SD
	Total # of colonies	94	121	114		
	%MF	4.3	5.0	4.4		
	**DATA**	**Trial 1**	**Trial 2**	**Trial 3**	**Ave**	**SD**
	G6* → A	0	0	0	0.0	0.0
	G6* → T	1	0	0	0.3	0.6
	G6* → C		1	0	0.5	0.7
	G6*/G7 del	3	4	5	4.0	1.0
	Off-target	0	1	0	0.3	0.6
	**% MUTATION FREQUENCY**					
	G6* → A	0.0	0.0	0.0	0.0	0.0
	G6* → T	1.1	0.0	0.0	0.4	0.6
	G6* → C	0.0	0.8	0.0	0.3	0.5
	G6*/G7 del	3.2	3.3	4.4	3.6	0.7
	Off-target	0.0	0.8	0.0	0.3	0.5
	Total %MF	4.3	5.0	4.4	4.5	0.4

SAMPLE	DATA	Trial 1	Trial 2	Trial 3	Ave	SD
**7-deaza 293T ZKO**	# of mutants	12	6	12	5.3	0.8
	# of WT	241	116	184		
	Total # of colonies	253	122	196		
	%MF	4.7	4.9	6.1		
	**DATA**	**Trial 1**	**Trial 2**	**Trial 3**	**Ave**	**SD**
	G6* → A	0	0	0	0.0	0.0
	G6* → T		0	0	0.0	0.0
	G6* → C	2		0	1.0	1.4
	G6*/G7 del	10	6	12	9.3	3.1
	Off-target	0		0	0.0	0.0
	**% MUTATION FREQUENCY**					
	G6* → A	0.0	0.0	0.0	0.0	0.0
	G6* → T	0.0	0.0	0.0	0.0	0.0
	G6* → C	0.8	0.0	0.0	0.3	0.5
	G6*/G7 del	4.0	4.9	6.1	5.0	1.1
	Off-target	0.0	0.0	0.0	0.0	0.0
	Total %MF	4.7	4.9	6.1	5.3	0.8

References

[1] S. Jiang, P. Li, C. C. Lai, J. A. Kelly, P. P. Roller. *J. Org. Chem*. **2006**, *71*, 7307-7314.

[2] F. Liu, J. Thomas, T. R. Burke Jr. *Synthesis* **2008**, 2432-2438.

[3] S. S. Pujari, Y. Zhang, S. Ji, M. D. Distefano, N. Y. Tretyakova. Chem. Commun. **2018**, *54*, 6296-6299.

[4] J. Wu, L. Li, P. Wang, C. You, N. L. Williams, Y. Wang. *Nucleic Acids Res.* **2016**, *44*, 9256-9265.

[5] H. Du, J. Leng, P. Li, L. Li, Y. Wang, *J. Biol. Chem.* **2018**, *293*, 11100-11108.
